# Mobile Genetic Element SCC*mec*-encoded *psm-mec* RNA Suppresses Translation of *agrA* and Attenuates MRSA Virulence

**DOI:** 10.1371/journal.ppat.1003269

**Published:** 2013-04-04

**Authors:** Chikara Kaito, Yuki Saito, Mariko Ikuo, Yosuke Omae, Han Mao, Gentaro Nagano, Tomoko Fujiyuki, Shunsuke Numata, Xiao Han, Kazuaki Obata, Setsuo Hasegawa, Hiroki Yamaguchi, Koiti Inokuchi, Teruyo Ito, Keiichi Hiramatsu, Kazuhisa Sekimizu

**Affiliations:** 1 Graduate School of Pharmaceutical Sciences, The University of Tokyo, Tokyo, Japan; 2 Department of Infection Control Science, Graduate School of Medicine, Juntendo University, Tokyo, Japan; 3 Sekino Hospital, Tokyo, Japan; 4 Sekino Clinical Pharmacology Clinic, Tokyo, Japan; 5 Division of Hematology, Department of Internal Medicine, Nippon Medical School, Tokyo, Japan; University of Tubingen, Germany

## Abstract

Community acquired-methicillin resistant *Staphylococcus aureus* (CA-MRSA) is a socially problematic pathogen that infects healthy individuals, causing severe disease. CA-MRSA is more virulent than hospital associated-MRSA (HA-MRSA). The underlying mechanism for the high virulence of CA-MRSA is not known. The transcription product of the *psm-mec* gene, located in the mobile genetic element SCC*mec* of HA-MRSA, but not CA-MRSA, suppresses the expression of phenol-soluble modulin α (PSMα), a cytolytic toxin of *S. aureus*. Here we report that *psm-mec* RNA inhibits translation of the *agrA* gene encoding a positive transcription factor for the PSMα gene *via* specific binding to *agrA* mRNA. Furthermore, 25% of 325 clinical MRSA isolates had a mutation in the *psm-mec* promoter that attenuated transcription, and 9% of the strains had no *psm-mec*. In most of these *psm-mec*-mutated or *psm-mec-*deleted HA-MRSAs, PSMα expression was increased compared with strains carrying intact *psm-mec*, and some mutated strains produced high amounts of PSMα comparable with that of CA-MRSA. Deletion of *psm-mec* from HA-MRSA strains carrying intact *psm-mec* increased the expression of AgrA protein and PSMα, and virulence in mice. Thus, *psm-mec* RNA suppresses MRSA virulence *via* inhibition of *agrA* translation and the absence of *psm-mec* function in CA-MRSA causes its high virulence property.

## Author Summary

Methicillin-resistant *Staphylococcus aureus* (MRSA) is resistant to various antibiotics, including β-lactams, thus causing serious clinical problems. Hospital-associated (HA)-MRSA infects immunocompromised patients in hospitals. Community-acquired (CA)-MRSA causes serious diseases in healthy people who have not had contact with hospitals in the United States, Canada, or Europe. CA-MRSA produces higher amounts of extracellular toxins and has higher virulence than HA-MRSA, although the reason for this is unclear. SCC*mec* is a foreign DNA integrated into the MRSA chromosome that contains several genes including the *mecA* gene that confers resistance against methicillin. The SCC*mec* of CA-MRSA does not contain the *psm-mec* gene that exists in the HA-MRSA SCC*mec*. In the present study, we found that the transcription product of *psm-mec* inhibits translation of the *agrA* gene encoding a positive transcription factor for many extracellular toxins by direct binding to the *agrA* mRNA, resulting in decreased extracellular toxin production. Furthermore, some HA-MRSA strains carry mutated *psm-mec* or no *psm-mec* and produce higher amounts of extracellular toxins than HA-MRSA strains carrying intact *psm-mec*. These findings suggest that *psm-mec* RNA negatively regulates *agrA* and mutation or absence of *psm-mec* leads to a high virulence capacity of MRSA.

## Introduction

CA-MRSA, especially the USA300 clone, causes severe infectious diseases in many people in the United States and in European countries. CA-MRSA is generally considered more virulent than most HA-MRSA [1]. Determining the molecular mechanism underlying the high virulence of CA-MRSA will be important toward establishing new therapeutic strategies against CA-MRSA infections. One reason for the high virulence of the CA-MRSA USA300 strains is suggested to be the high amounts of secreted toxins, including PSMα, α-hemolysin, δ-hemolysin (Hld), and the Panton-Valentine leukocidin (PVL) [1], [2], [3]. The USA300 strains show increased expression of the *agr* locus, which upregulates the production of PSMα, α-hemolysin, and PVL, compared with HA-MRSA strains [1], [3], [4], [5]. The *agr* locus is essential for the virulence of the USA300 strains against animals [4], [6]. The *agr* locus encodes *agrBDCA*, which functions in quorum sensing [7]. An extracellular quorum-sensing molecule made from AgrD activates the sensor protein AgrC. AgrC activates the transcription factor AgrA by phosphorylation. AgrA activates the transcription of *agrBDCA*, including *agrA* itself [8]. Thus, quorum sensing is under positive feedback regulation. The *agr* locus also encodes RNAIII, which is an mRNA of Hld as well as a regulatory RNA that upregulates the expression of various toxins, including α-hemolysin, and downregulates the expression of various cell surface proteins [9]. AgrA activates the transcription of RNAIII and other virulence genes, including the *psmα* operon, by direct binding to the promoter [8], [10]. The mechanism that increases the expression of *agr* in the USA300 strains, however, is not known.

SCC*mec* is a mobile genetic element that confers methicillin resistance to MRSA strains. The structure of the SCC*mec* region differs between the CA-MRSA and HA-MRSA strains [11]. We previously reported that the *psm-mec* gene that exists in type-II and type-III SCC*mec*, which is found in most HA-MRSA, regulates the virulence of *S. aureus* [12]. The *psm-mec* gene is absent in type-IV SCC*mec* of the CA-MRSA USA300 strains [12]. Introduction of *psm-mec* into FRP3757, a CA-MRSA USA300 strain, or Newman, a methicillin-sensitive *S. aureus* strain that carries neither SCC*mec* nor *psm-mec*, decreases the secreted amount of PSMα, suppresses colony-spreading ability, and promotes biofilm formation [12], [13]. Furthermore, the *psm-mec*-transformed strains attenuate virulence in a mouse model of systemic infection [12], [13]. The finding first revealed that a factor encoded by a mobile-genetic element negatively regulates bacterial virulence. Because the USA300 strains have no *psm-mec*, we proposed that the absence of *psm-mec* is a genetic determinant of the high virulence of USA300 [12]. Suppression of colony-spreading and promotion of biofilm formation by the *psm-mec* gene are attributed to both the transcription product and the translation product of *psm-mec* [12]. In contrast, suppression of the expression of PSMα is attributed to the transcription product of *psm-mec*, i.e., *psm-mec* RNA [12]. An mRNA encoding protein rarely acts as a regulatory RNA. Such bifunctional RNA, other than *psm-mec* RNA, is found as *S. aureus* RNAIII [9] and *Escherichia coli* SgrS [14], and is important material for investigations of gene evolution. In this study, to clarify the molecular mechanisms underlying the inhibitory effect of *psm-mec* RNA on PSMα expression, we screened and identified a target molecule of *psm-mec* RNA. Additionally, we performed an epidemiologic study and a deletion analysis of *psm-mec* to verify whether *psm-mec* mutation confers a high virulence property to MRSA strains.

## Results and Discussion

### *psm-mec* RNA inhibits *agrA* translation

**Figure 1:**
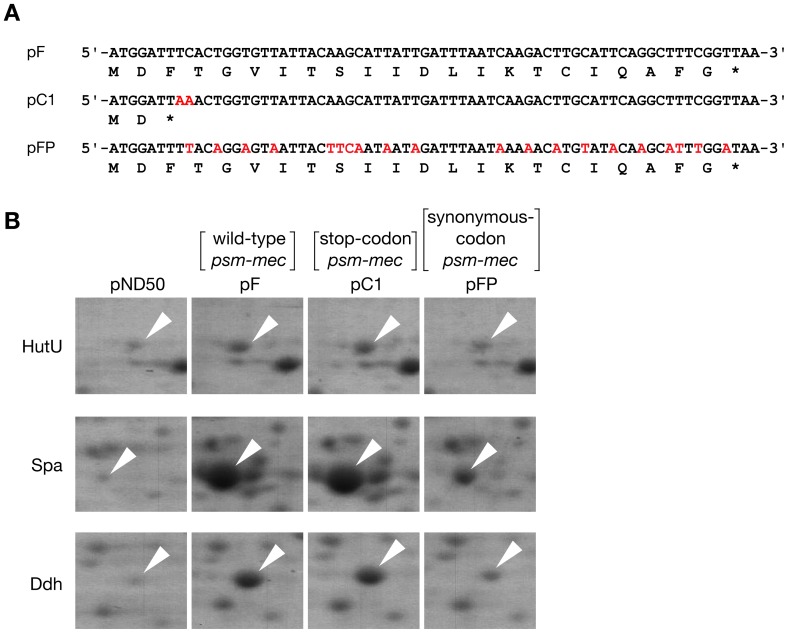
*psm-mec* RNA increased the amount of HutU, Spa, and Ddh in CA-MRSA FRP3757 (USA300). (**A**) The nucleotide sequence of the *psm-mec* ORF in pF, the stop-codon introduced sequence of *psm-mec* ORF in pC1, and the synonymous-codon substituted sequence of *psm-mec* ORF in pFP are shown. The substituted nucleotides are colored in red. The amino acid sequence of PSM-mec protein is shown below the respective nucleotide sequence. (**B**) Cell extract of FRP3757 strain that was transformed with empty vector (pND50), *psm-mec* (pF), mutated *psm-mec* harboring a stop codon (pC1), or mutated *psm-mec* harboring synonymous codon substitutions (pFP) was analyzed by two-dimensional electrophoresis. Proteins were stained with Coomassie Brilliant Blue. The protein spot was excised and identified by matrix-assisted laser desorption ionization time-of-flight mass spectrometry (**Table S1**).

**Figure 2:**
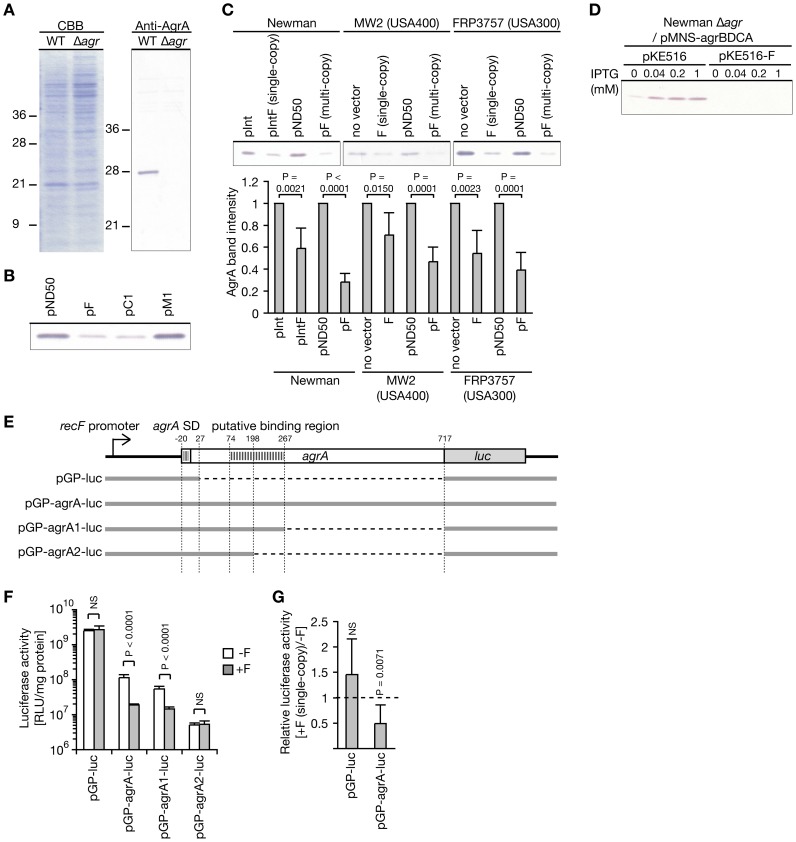
*psm-mec* RNA inhibits *agrA* translation. (**A**) Cell extracts of overnight cultures of Newman strain (WT) and the *agr-*null mutant (Δ*agr*) were electrophoresed in sodium dodecyl sulfate-polyacrylamide gels. One gel was stained with Coomassie Brilliant Blue (Left panel). Proteins in another gel were transferred to a membrane and used for Western blotting by anti-AgrA IgG (Right panel). (**B**) Cell extracts of 24 h-cultures of Newman strains transformed with empty vector (pND50), a plasmid carrying wild-type *psm-mec* (pF), a plasmid carrying *psm-mec* with a stop-codon (pC1), and a plasmid carrying *psm-mec* with the -7T>C promoter mutation (pM1) were subjected to Western blotting by anti-AgrA IgG. Each lane contains 3.5 µg proteins of cell extracts. (**C**) Cell extracts of 24 h-cultures of Newman, MW2 (USA400), and FRP3757 (USA300) strains that were transformed with pF carrying *psm-mec* (multi-copy), or integrated with *psm-mec* into the chromosome (single-copy) were subjected to Western blotting by anti-AgrA IgG (Upper panel). Each lane contains 3 µg proteins of cell extracts. Band intensities of AgrA were measured and are presented in the lower graph. The vertical axis represents the relative value against the AgrA band intensity of the parent strain in each Newman, MW2, and FRP3757 genetic background. Means ± standard deviations from four independent experiments are presented. Student t-test P-values between the parent strain and the *psm-mec*-introduced strain in each genetic background are presented. (**D**) The *agr* null mutant of Newman transformed with pMNS-agrBDCA carrying IPTG-inducible *agrBDCA* and pKE516 (empty vector), or pMNS-agrBDCA and pKE516-F carrying wild-type *psm-mec* was cultured in the presence or absence of IPTG. Cell extracts of 24-h cultures were subjected to Western blotting by anti-AgrA IgG. Each lane contains 6 µg proteins of cell extracts. (**E**) Schematic representation of *luc-*fusions of the *recF* promoter, *agrA* SD, the *agrA* ORF, and the *luc* ORF. Bold gray lines represent the plasmid construct. Horizontal dotted lines represent the regions deleted from the plasmids. Putative binding region means the region predicted to bind to the *psm-mec* RNA by *in silico* analysis. SD means Shine-Dalgarno sequence of *agrA*. (**F**) Luciferase activities of Newman strains that were transformed with the *luc-*fusion plasmids with *psm-mec* (+F) or without *psm-mec* (−F) were measured. The vertical axis represents the luciferase activity. Student t-test P-values between +F and −F are presented. NS, P>0.05. (**G**) Newman strain, which was integrated with *psm-mec* or without *psm-mec*, was transformed with the *luc-*fusion plasmids. Luciferase activities of the strains were measured. The vertical axis represents the relative luciferase activity of the *psm-mec*-integrated Newman [+F (single-copy)] against that of the Newman strain (−F). Student t-test P-values between +F and −F are presented. NS, P>0.05.

First, to identify the target molecule of *psm-mec* RNA, we compared the protein expression patterns of the CA-MRSA FRP3757 strain (USA300) and its transformant with *psm-mec* by two-dimensional gel electrophoresis. In the *psm-mec* (pF)-transformed FRP3757, the expression of HutU, protein A, and Ddh were increased (**Fig. 1** **, Table S1**). The increases in the amounts of the proteins were not diminished by the introduction of a stop-codon into the third codon of *psm-mec* open reading frame (ORF), but they were diminished by the introduction of synonymous codons into the *psm-mec* ORF, which altered the RNA nucleotide sequence but not the amino acid sequence (**Fig. 1**). The mutated *psm-mec* containing a stop-codon (pC1) expresses *psm-mec* RNA without the expression of PSM-mec protein [12]. The mutated *psm-mec* harboring synonymous codon substitutions (pFP) expresses PSM-mec protein [12]. Thus, we concluded that *psm-mec* RNA increased the expression of these proteins. Previous reports indicated that these three proteins are downregulated by the *agr* locus [15], [16], [17]. We previously found that *psmα* promoter activity, which is enhanced by AgrA, was decreased in the Newman strain transformed with *psm-mec*, resulting in a decreased amount of PSMα, but no alteration of the amount of *agrA* mRNA [12]. Based on these findings, we hypothesized that the introduction of *psm-mec* leads to the inhibition of the translation of *agrA* and decreases the amount of AgrA in the cells. To examine this possibility, we first established a method to determine the amount of AgrA protein in cells. Anti-AgrA immunoglobulin (IgG) was prepared from a rabbit immunized with His-tagged recombinant AgrA and Western blot analysis was performed. In cell extracts of the Newman strain, the 28-kDa band of AgrA was detected, which was consistent with the predicted molecular mass of AgrA, 27.9 kDa (**Fig. 2A**). In contrast, the band was not detected in cell extracts of the *agr-*null mutant (**Fig. 2A**). In addition, the band was not detected using IgG from a non-immunized rabbit (data not shown). Therefore, we concluded that the 28-kDa protein detected by anti-AgrA IgG was the AgrA protein. We then performed Western blot analysis of AgrA in *psm-mec*-transformed Newman strain. In the *psm-mec* (pF)*-*transformed Newman strain, the AgrA band intensity was decreased compared with the vector (pND50)-transformed Newman strain (**Fig. 2B**). Decreased intensity of the AgrA band was also observed in Newman transformed with *psm-mec* containing a stop-codon (pC1) (**Fig. 2B**), which expresses *psm-mec* RNA without the expression of PSM-mec protein [12]. In contrast, the *psm-mec* gene, which has a -7T>C mutation in the promoter (pM1) and does not express *psm-mec* RNA [12], did not decrease the AgrA band intensity (**Fig. 2B**). These findings suggest that the transcription product of *psm-mec* acts to decrease the amount of AgrA in *S. aureus* cells. Furthermore, we examined whether *psm-mec* RNA also decreases the amount of AgrA in the CA-MRSA strains MW2 (USA400) and FRP3757 (USA300). In both CA-MRSA strains, the introduction of a plasmid carrying *psm-mec* (pF) led to a decrease in the amount of AgrA (**Fig. 2C**). We further analyzed whether a single copy of *psm-mec* is enough to decrease the amount of AgrA in respective *S. aureus* strains. We previously reported the construction of a Newman strain integrated with *psm-mec* into the chromosomal DNA [13]. In this study, we constructed MW2 and FRP3757 strains into which *psm-mec* was integrated into the chromosomal regions near the *mecA* of SCC*mec*, where *psm-mec* is originally present in HA-MRSA strains (**Fig. S1**). In these three strains, integration of *psm-mec* into the chromosome decreased the amount of AgrA in the cells (**Fig. 2C**). These findings indicate that introduction of *psm-mec* into CA-MRSA strains decreases the amount of AgrA and that a single copy *psm-mec* is enough to exert the repression effect.

Next, we examined whether *psm-mec* represses the translation of *agrA*. AgrA functions in a positive feedback loop to activate the transcription of *agrBDCA*, including *agrA* itself. To exclude the effect on the transcription initiation of *agrBDCA* from its native promoter, we transformed the *agr-*null mutant of Newman with pMNS-agrBDCA, which expresses *agrBDCA* from an IPTG-inducible promoter. The amount of AgrA was increased by increasing the IPTG concentration in the strain transformed with pMNS-agrBDCA and empty vector (pKE516), whereas the introduction of a plasmid carrying *psm-mec* (pKE516-F) into the strain transformed with pMNS-agrBDCA diminished AgrA expression in the presence of IPTG (**Fig. 2D**). Thus, *psm-mec* inhibits AgrA expression without the transcriptional regulation of the *agrBDCA* promoter. To examine the effect of *psm-mec* on the *agrA* coding region, we constructed a reporter gene-fusion construct in which the Shine-Dalgarno sequence of *agrA* and *agrA* ORF was fused in frame with *luc* under the control of the *recF* promoter (**Fig. 2E**). The introduction of *psm-mec* did not decrease the luciferase activity of *luc-*fusion with the −20–+27 sequence of *agrA* (pGP-luc), although it decreased the luciferase activity of *luc*-fusion with the −20–+717 sequence of *agrA* (pGP-agrA-luc) (**Fig. 2F**). Decreased luciferase activity of pGP-agrA-luc was also observed in Newman integrated with a single copy of *psm-mec* (**Fig. 2G**). These findings indicate that *psm-mec* inhibits the translation of *agrA*.

### *psm-mec* RNA specifically binds *agrA* mRNA

**Figure 3:**
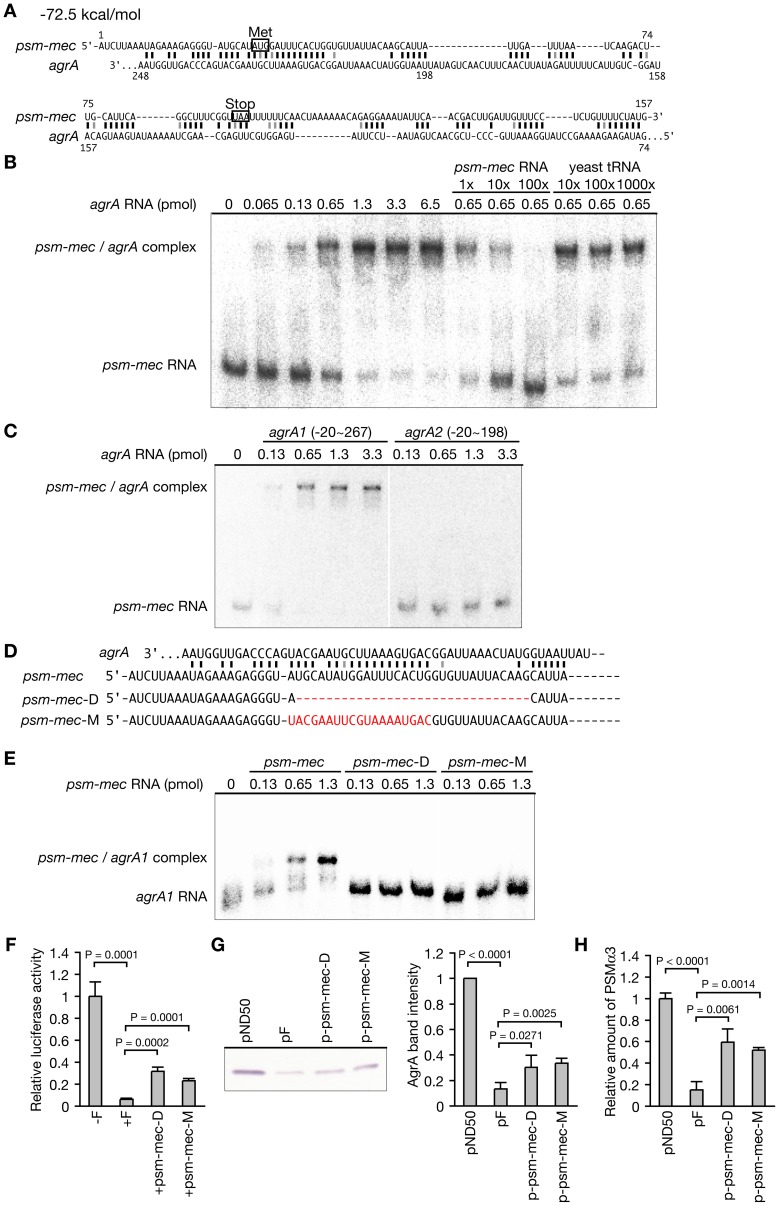
*psm-mec* RNA specifically binds *agrA* mRNA and inhibits its translation. (**A**) Hybridization between *psm-mec* RNA and *agrA* mRNA was predicted by an *in silico* program RNA hybrid. Black and gray lines represent strong and weak hydrogen bonds, respectively. (**B**) Binding between *psm-mec* RNA and *agrA* RNA (−20–717) was analyzed using a gel-retardation assay. Various amounts of nonlabeled *agrA* RNA were added to ^32^P-labeled *psm-mec* RNA (0.13 pmol), and electrophoresed in 6% native polyacrylamide gel. In the right six lanes, nonlabeled *psm-mec* RNA or yeast tRNA was added to compete with the binding between *agrA* RNA and ^32^P-labeled *psm-mec* RNA. (**C**) Binding experiment between *psm-mec* RNA and deletion mutants of *agrA* RNA. Various amounts of nonlabeled *agrA1* RNA (−20–267) or *agrA2* RNA (−20–198) were added to ^32^P-labeled *psm-mec* RNA (0.13 pmol). (**D**) Nucleotide sequences of *psm-mec* RNA, a deletion mutant of *psm-mec* RNA (*psm-mec-*D), and a nucleotide-substituted *psm-mec* RNA (*psm-mec-*M) are presented. Red dotted line in *psm-mec*-D indicates the deleted region. Red letters in *psm-mec-*M indicate the substituted nucleotides that are not complementary to *agrA* RNA. (**E**) Various amounts of nonlabeled *psm-mec* RNA, *psm-mec-*D RNA, or *psm-mec-*M RNA were added to ^32^P-labeled *agrA1* RNA (−20–267), and electrophoresed in 6% native polyacrylamide gel. (**F**) Luciferase activities of Newman strains that were transformed with pGP-agrA-luc carrying no *psm-mec* (−F), *psm-mec* (+F), *psm-mec*-D, or *psm-mec*-M were measured. The vertical axis represents the relative luciferase activity against that of pGP-agrA-luc carrying no *psm-mec*. Means ± standard deviations from three independent experiments are presented. Student t-test P-values are presented. (**G**) Cell extracts (3 µg protein) of 24 h-cultures of Newman strains transformed with pND50 (empty vector), pF carrying *psm-mec*, p-psm-mec-D carrying *psm-mec-*D, or p-psm-mec-M carrying *psm-mec-*M were subjected to Western blotting by anti-AgrA IgG (Left panel). Band intensities of AgrA were measured (Right graph). Means ± standard deviations from three independent experiments are presented. Student t-test P-values are presented. (**H**) Amounts of PSMα3 in the supernatants of 24 h-cultures of Newman strains transformed with *psm-mec*, *psm-mec-*D, or *psm-mec*-M were measured. The vertical axis represents the relative amount of PSMα3 against that of Newman strain transformed with pND50 (empty vector). Student t-test P-values are presented.

We next searched for mRNA interacting with *psm-mec* RNA using the *in silico* programs sRNATarget [18] and RNAhybrid [19], and identified *agrA* mRNA as a candidate (**Fig. 3A**). We hypothesized that the inhibition of *agrA* translation by *psm-mec* is caused by the direct binding of *psm-mec* RNA to *agrA* mRNA. Primer extension [12] and nuclease S1 protection analyses (**Fig. S2**) revealed that the size of *psm-mec* RNA was 157 bases. To examine the direct binding of *psm-mec* RNA to *agrA* mRNA, we performed a gel shift analysis using 157 bases of *psm-mec* RNA that was synthesized by *in vitro* transcription. The addition of the −20–+717 sequence of *agrA* mRNA retarded the mobility of the radiolabeled *psm-mec* RNA fragment in a dose-dependent manner (**Fig. 3B**). The retardation was cancelled by the addition of nonlabeled *psm-mec* RNA, although not by the addition of a 1000-fold amount of yeast tRNA (**Fig. 3B**). Thus, the binding of *psm-mec* RNA to *agrA* mRNA detected by gel shift assay was specific. Furthermore, to identify the region of *agrA* mRNA required for binding to *psm-mec* RNA, we examined whether the −20–+267 sequence of *agrA* mRNA (*agrA1*), which contains the binding regions predicted by *in silico* analysis; the +74-+267 sequence of *agrA*, and the −20–+198 sequence of *agrA* mRNA (*agrA2*), which partially disrupts the predicted binding region, bind *psm-mec* RNA. This finding demonstrated that *agrA1* RNA binds *psm-mec* RNA, although *agrA2* RNA does not bind *psm-mec* RNA (**Fig. 3C**). Thus, the +199-+267 sequence of *agrA* mRNA is required for binding to *psm-mec* RNA. To determine whether the binding of *psm-mec* RNA to *agrA* mRNA is required for the inhibition of *agrA* translation, we performed a reporter gene-fusion analysis with and without the +199-+267 region of *agrA*. We compared the luciferase activity of *luc*-fusions with *agrA1* that can bind *psm-mec* RNA (pGP-agrA1-luc) and *agrA2* that cannot bind *psm-mec* RNA (pGP-agrA2-luc) (**Fig. 2E**). The expression of *agrA1-luc* was suppressed by *psm-mec* in a similar manner as *agrA-luc*, although the expression of *agrA2-luc* was not suppressed by *psm-mec* (**Fig. 2F**). These findings suggest that the binding of *psm-mec* RNA to *agrA* mRNA leads to the inhibition of *agrA* translation.

To identify the region of *psm-mec* RNA that is required for binding *agrA* mRNA, we constructed a *psm-mec-*D disrupting 22–52 sequence that is a partial region of the *in silico* predicted binding region and a *psm-mec-*M carrying a mutated 21–39 sequence, which are not complementary to *agrA* mRNA (**Fig. 3D**). Wild-type *psm-mec* RNA retarded the migration of *agrA1* RNA, whereas these mutated *psm-mec* RNA did not retard the migration of *agrA1* RNA (**Fig. 3E**). Thus, the 21–52 sequence of *psm-mec* RNA is required for binding to *agrA* mRNA. To verify whether binding of *psm-mec* RNA to *agrA* mRNA is required to repress the translation of *agrA* and inhibit PSMα3 expression by *psm-mec*, we examined the effect of *psm-mec*-D and *psm-mec*-M on the activity of *agrA-*luc fusion, the amount of AgrA, and the expression of PSMα3. The luciferase activity of *agrA-*luc fusion was partially restored in the presence of *psm-mec-*D or *psm-mec*-M compared with wild-type *psm-mec* (**Fig. 3F**). The amount of AgrA was partially relieved in Newman transformed with *psm-mec-*D or *psm-mec-*M compared with that in Newman transformed with wild-type *psm-mec* (pF), which inhibited the expression of AgrA (**Fig. 3G**). The expression of PSMα3 was repressed by wild-type *psm-mec*, whereas the repression effect was attenuated in *psm-mec-*D or *psm-mec-*M (**Fig. 3H**). Therefore, binding of *psm-mec* RNA to *agrA* mRNA is required to repress the translation of *agrA* and to inhibit PSMα3 expression by *psm-mec*.

**Figure 4:**
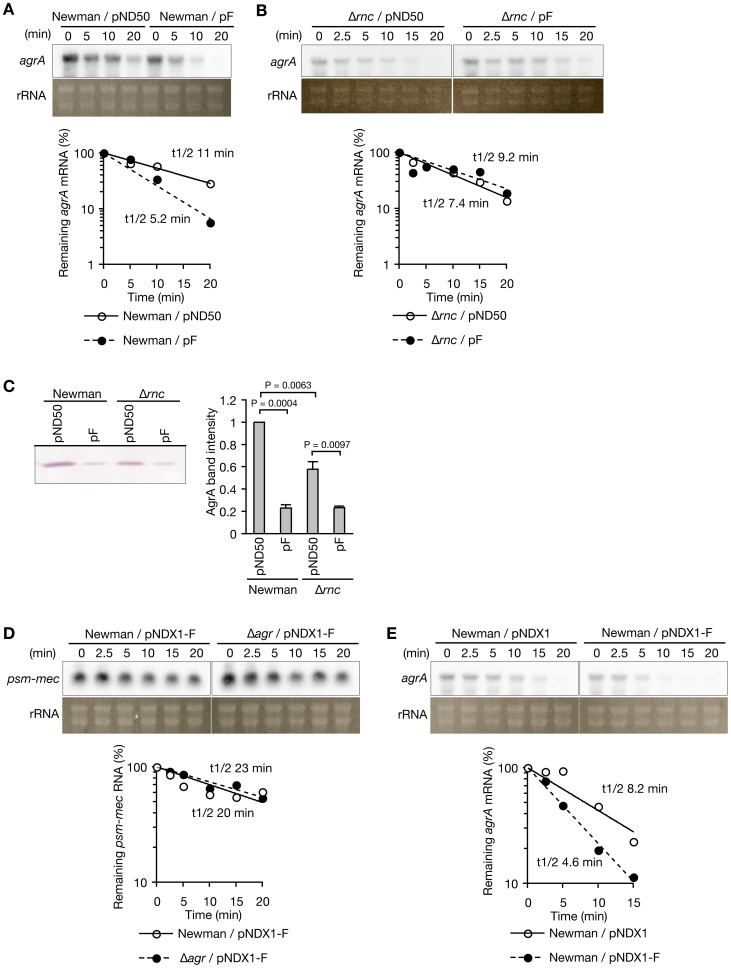
*psm-mec* affects the stability of *agrA* mRNA. (**A**) Northern blot analysis was performed to measure *agrA* mRNA stability in Newman strain transformed with an empty vector (pND50) or pF carrying *psm-mec*. Total RNA was extracted from cultures (A_600_ = 3) at the indicated time point after rifampicin treatment. *agrA* mRNA (RNAII) was detected by^32^P-labeled DNA probe. rRNA was stained with ethidium bromide. The amounts of *agrA* mRNA were normalized with the amount of 16S rRNA at each time-point and the amounts of *agrA* mRNA relative to the amount at 0 min are shown in graph. The half-life at which 50% of *agrA* mRNA remained was determined by exponential approximation. Data are representative from three independent experiments. (**B**) *agrA* mRNA stability was measured in the *rnc-*deleted mutant transformed with pND50 or pF. Total RNA was extracted from cultures (A_600_ = 3) at the indicated time point after rifampicin treatment. Data presentation and the calculation of the RNA half-life are the same as in (A). Data are representative from three independent experiments. (**C**) Cell extracts (4.2 µg protein) of 24-h cultures of Newman strains transformed with pND50 or pF and the *rnc-*deleted mutant transformed with pND50 or pF were subjected to Western blotting by anti-AgrA IgG (Left panel). Band intensities of AgrA were measured (Right graph). Means ± standard deviations from two independent experiments are presented. Student t-test P-values are presented. (**D**) *psm-mec* RNA stability was measured in Newman and the *agr-*null mutant, which were transformed with anhydrotetracycline-inducible *psm-mec* (pNDX1-F). *S. aureus* cells were grown to A_600_ = 2 in the presence of 0.4 µg/ml of anhydrotetracycline. Total RNA was extracted after rifampicin treatment and electrophoresed. Data presentation and the calculation of the RNA half-life are the same as in (A). Data are representative from two independent experiments. (**E**) *agrA* mRNA stability was measured in Newman transformed with empty vector (pNDX1) or anhydrotetracycline-inducible *psm-mec* (pNDX1-F). *S. aureus* cells were grown to A_600_ = 2 in the presence of 0.4 µg/ml of anhydrotetracycline. Total RNA was extracted after rifampicin treatment and electrophoresed. Data presentation and the calculation of the RNA half-life are the same as in (A). Data are representative from two independent experiments.

Translational inhibition of *agrA* by *psm-mec* might be due to the destabilization of *agrA* mRNA by *psm-mec*. We examined the stability of *agrA* mRNA in the presence or absence of *psm-mec*. In the vector (pND50)-transformed Newman strain, the half-life of *agrA* mRNA was 11 min, whereas in the *psm-mec* (pF)-transformed Newman strain, the half-life of *agrA* mRNA was 5 min (**Fig. 4A**). Thus, *psm-mec* slightly decreased the stability of *agrA* mRNA. RNase III is an endoribonuclease that catalyzes double-stranded RNA and contributes to repress gene expression by the regulatory RNA, RNAIII [20], [21], [22]. We examined whether RNase III encoded by the *rnc* gene contributes to the alteration of the half-life of *agrA* mRNA by *psm-mec*. In the *rnc*-deleted mutant, introduction of *psm-mec* (pF) did not decrease the half-life of *agrA* mRNA compared with introduction of the vector (pND50) (**Fig. 4B**). Therefore, RNase III is required for the destabilization of *agrA* mRNA by *psm-mec*. We then examined the inhibitory effect of *psm-mec* on the translation of *agrA* under the *rnc*-deletion background, in which *psm-mec* did not decrease the stability of *agrA* mRNA. In the *rnc-*deleted mutant, introduction of *psm-mec* (pF) decreased the amount of AgrA (**Fig. 4C**). These results suggest that *psm-mec* represses the translation of *agrA* independently of the decrease in stability of *agrA* mRNA, although *psm-mec* acts to decrease the stability of *agrA* mRNA *via* RNase III. Next, we examined whether the stability of *psm-mec* RNA was affected by *agrA*. *psm-mec* RNA was expressed from an anhydrotetracycline-inducible promoter, because *psm-mec* transcription is positively regulated by AgrA [23]. There was no difference in the half-life of *psm-mec* RNA between Newman strain and the *agr*-null mutant and the half-life was approximately 20 min (**Fig. 4D**). In this condition, *psm-mec* RNA slightly decreased the stability of *agrA* mRNA, which is consistent with the finding using *psm-mec* expressed from the native promoter (**Fig. 4E**). These results suggest that *psm-mec* RNA is stable and the stability is not affected by *agrA* mRNA.

### Mutation or absence of *psm-mec* correlates with high expression of extracellular PSMα in MRSA clinical isolates

**Figure 5:**
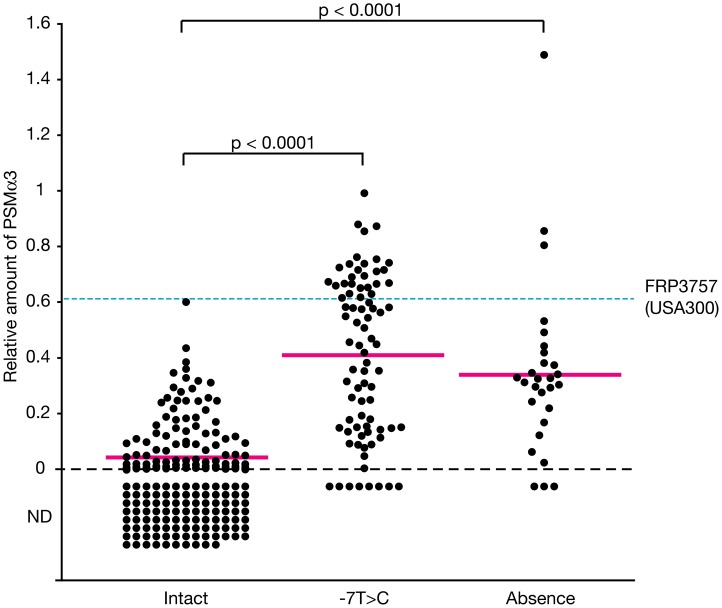
MRSA clinical isolates harboring a *psm-mec* mutation produce high amounts of PSMα3. Nucleotide sequences of *psm-mec* genes of 325 MRSA isolates were determined (**Table 1**). MRSA strains harboring intact *psm-mec* (Intact), -7T>C-mutated *psm-mec* (-7T>C), or no *psm-mec* (Absence) were cultured for 15 h. The amounts of PSMα3 in the culture supernatants were measured. The vertical axis represents the relative amount of PSMα3 against that of Newman strain. Closed circles represent the amounts of PSMα3 of each MRSA strains, which are the means from two independent experiments. Magenta lines represent the averaged amount of PSMα3 of each MRSA groups. Cyan dotted line represents the amount of PSMα3 of CA-MRSA strain FRP3757 (USA300). Student t-test P-values are presented. ND, not detected.

Because *psm-mec* inhibits *agrA* translation, resulting in the repression of PSMα3 expression, we hypothesized that the *psm-mec* mutation was related to the high expression levels of PSMα3 in clinical MRSA isolates. We collected 325 clinical MRSA strains from three hospitals in the Kanto area in Japan, and sequenced their *psm-mec* genes. Eighty-one strains (25%) carried the -7T>C mutation in the *psm-mec* promoter and one strain carried an insertion of 2.2 kbp and the -4G>A mutation in the *psm-mec* promoter, both of which repressed the expression of *psm-mec* in the Newman strain (**Table 1**). Twenty-eight strains (9%) did not carry *psm-mec* (**Table 1**). As we reported previously [12], -7T>C-mutated *psm-mec* lacked the ability to inhibit PSMα3 expression and colony spreading, and to promote biofilm formation in the Newman strain (**Fig. S3**). The *psm-mec* carrying an insertion of 2.2 kbp and the -4G>A mutation also lacked these abilities (**Fig. S3**). In contrast, in other *psm-mec* mutations, such as D2, D4, and D5, which did not decrease the expression of *psm-mec*, the inhibitory abilities of PSMα3 expression and colony spreading, and the promotion of biofilm formation in the Newman strain were maintained (**Fig. S3**). We next examined whether 81 strains carrying -7T>C *psm-mec* and 28 strains without *psm-mec* had higher amounts of PSMα3 than the other 193 strains carrying intact *psm-mec*. The amount of PSMα3 in the culture supernatant of each strain was determined by high performance liquid chromatography analysis. The findings revealed that these strains carrying mutations in *psm-mec* expressed higher amounts of PSMα3 in the culture supernatant than the strains carrying intact *psm-mec* (**Fig. 5**). Some strains carrying the mutations produced higher amounts of PSMα3 than the CA-MRSA FRP3757 strain (USA300) (**Fig. 5**). These findings suggest that the *psm-mec* mutations increase the amount of PSMα3 in HA-MRSA isolates, and that there are some strains in the *psm-mec-*mutated isolates that produce even higher amounts of PSMα3 than produced by CA-MRSA.

**Table 1:** Identification of mutations of the *psm-mec* gene from MRSA strains.

Name	Mutation of *psm-mec*	Expression (%)	Number of isolates	%
D1	-7T>C	0	81	25
D2	-42A>G	150	3	1
D3	−70–71 insertion of 2.2 kbp^1^; -4G>A	0	1	0
D4	−74–75 insertion of T	125	18	6
D5	−242–243 insertion of 1.3 kbp^2^	70	1	0
Absence	no *psm-mec*	0	28	9
Intact	intact *psm-mec*	100	193	59
Total			325	100

Mutation of *psm-mec* is presented as a number of nucleotides from the transcription start site of *psm-mec* and nucleotide substitutions. T>C means that thymine was exchanged with cytosine. Expression of the respective mutated *psm-mec* gene in the Newman strain was examined (**Fig. S3**) and is presented in the column ‘Expression’. 1, DNA fragment of 2206 bp (GenBank, AB 729111). 2, DNA fragment of 1332 bp (GenBank, AB 729110).

To investigate whether the genetic backgrounds of the clinical isolates carrying -7T>C *psm-mec* or no *psm-mec* differ from those carrying intact *psm-mec*, we determined the SCC*mec* types and *spa* types of all tested strains. One hundred and twenty-seven strains (65%) of the isolates carrying intact *psm-mec* had type II SCC*mec* (**Table 2**). Seventy-five of 81 strains (93%) carrying -7T>C *psm-mec* had type II SCC*mec* (**Table 2**). The results of SCC*mec* typing were in high agreement with the previously reported data that *psm-mec* is closely related to the class A *mec* gene complex carried by either types II, III, or VIII of the SCC*mec* element [13], [23], [24]. Most of the strains carrying intact *psm-mec* or -7T>C *psm-mec* had type 2 *spa*: 122 (63%) of the isolates carrying intact *psm-mec*; 69 (85%) of the isolates carrying -7T>C *psm-mec* (**Table 3**). In contrast, all isolates not carrying *psm-mec* also did not carry class A *mec* and the majority of them carried either type I or IV SCC*mec* elements (**Table 2**). Sixteen strains (55%) of the isolates not carrying *psm-mec* had type 855 *spa* and three strains (10%) had type 2 *spa* (**Table 3**). These results indicate that most isolates carrying -7T>C *psm-mec* and intact *psm-mec* have closely related genetic backgrounds, whereas most isolates not carrying *psm-mec* have different genetic backgrounds compared to isolates carrying intact or -7T>C *psm-mec*. In addition, there are various *spa* types in the isolates carrying -7T>C *psm-mec* or no *psm-mec*, indicating that the isolates carrying the *psm-mec* mutation are polyclonal.

**Table 2:** Typing of SCC*mec* of MRSA clinical isolates.

		Number of isolates belonging to each SCC*mec* type							
		SCC*mec* type	II	n.a.^1^	IV	I	n.a.	n.a.	NT^3^
		*ccr* type	2	2+5	2	1	2	2+4	
Name	Total^2^	*mec* class	A	A	B	B	C2	A	
D1 (-7T>C)	81		75						6
D2	3		2						1
D3	1								
D4	18		14	2					2
D5	1								
Absence	28				21	1	1		5
Intact	193		127	48				2	16

*ccr* genes and *mec* gene complex were identified by multiplex PCRs [48]. All isolates were *mecA* positive. SCC*mec* types, I, II, and IV were assigned by the combination of types of *ccr* gene and *mec* gene complex. Abbreviations are as follows:

1 n.a., SCC*mec* type could not be assigned from the experiments;

2 Total, total number of isolates;

3 NT, non-typed, since DNA fragment was not amplified by PCR identifying either *ccr* genes or *mec* gene complex. ‘2+5’ in *ccr* type means that both type 2 and type 5 *ccr* were identified, indicating that 48 strains (25%) carry type II SCC*mec* and SCC carrying *ccrC*. ‘2+4’ in *ccr* type indicates that 2 strains (1%) carry type II or type VIII SCC*mec*. The combination of type 2 *ccr* and class C2 *mec* gene complex suggests that it might be a novel SCC*mec* element. Since it was out of scope of this paper, we classified it in the group of not assigned.

**Table 3:** Typing of *spa* of MRSA clinical isolates.

		Number of isolates belonging to each *spa* type																									
Name	Total	2	929	693	387	337	26	248	416	513	14	29	45	23	410	230	339	268	1178	696	606	385	855	222	799	143	New
D1 (-7T>C)	81	69	3	3	3	1		1																			1
D2	3	3																									
D3	1								1																		
D4	18	17						1																			
D5	1	1																									
Absence	28	3							1												3	1	16	1	1	1	1
Intact	193	122					49			3	2	4	2	2	1	1	1	1	1	1							3

*spa* types were identified by sequencing short-sequence repeats (SSRs) of *spa* gene [47]. ‘New’ means new *spa* types that were identified in this study. These *spa* types were assigned as *spa* types 1491, 1492, 1493, and 1494.

### *psm-mec* is required for suppression of the virulence in MRSA clinical isolates

**Figure 6:**
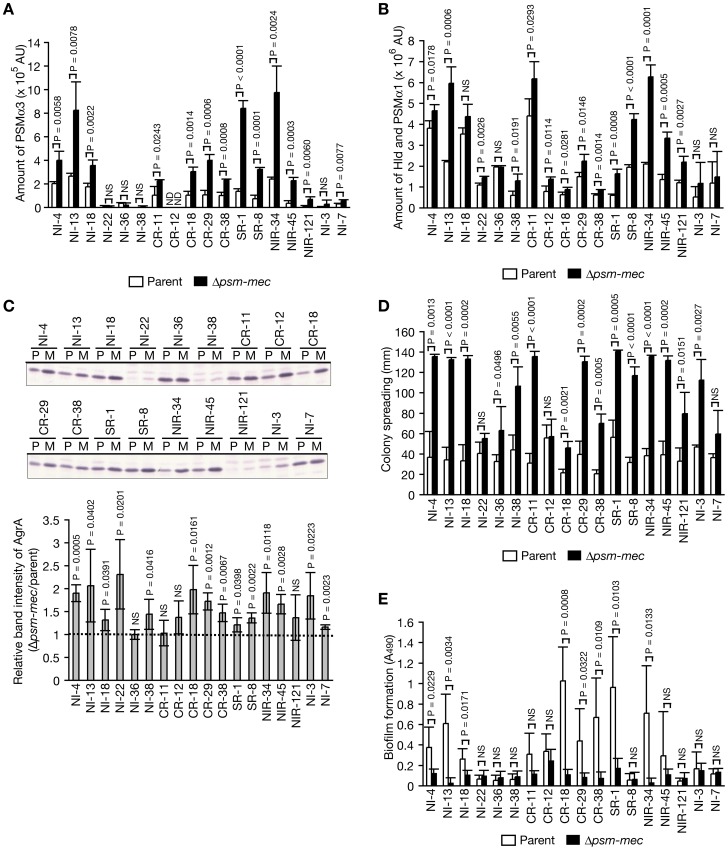
Deletion of *psm-mec* in MRSA clinical isolates increases the PSMα production, *agrA* expression, and colony spreading, whereas decreases biofilm formation. (**A, B**) The amounts of PSMα3 (A) and Hld + PSMα1 (B) of 18 MRSA isolates and its *psm-mec-*deleted mutants were measured. White bar represents the clinical isolate used as the parent strain. Black bar represents the *psm-mec*-deleted mutant of the clinical isolate. The vertical axis represents the amount of PSMαs in arbitrary units based on A_215_. Means ± standard deviations from three independent experiments are shown. Student t-test P-values between the parent strain and the *psm-mec*-deleted mutant are presented. NS, P>0.05. (**C**) Cell extracts (3.7 µg protein) of 15 h-cultures of clinical MRSA isolates and the *psm-mec*-deleted mutants were subjected to Western blotting by anti-AgrA IgG (Upper panel). Band intensities of AgrA were measured and are presented as relative values against that of the parent strain (Lower graph). Means ± standard deviations from three independent experiments are presented. Student t-test P-values between the parent strain and the *psm-mec*-deleted mutant are presented. NS, P>0.05. (**D**) Colony spreading abilities of clinical MRSA isolates and the *psm-mec*-deleted mutants were evaluated. Overnight cultures were spotted onto soft agar plates and incubated for 24 h at 37°C. The vertical axis represents diameters of giant colonies. Means ± standard deviations from three independent experiments are shown. Student t-test P-values between parent strain and the *psm-mec*-deleted mutant are presented. NS, P>0.05. (**E**) Biofilm formation of clinical MRSA isolates and *psm-mec*-deleted mutants were evaluated. Bacterial strains were grown on polystyrene plates for 3 days and the biofilm amounts were measured. White bar represents the clinical isolate used as the parent strain. Black bar represents the *psm-mec*-deleted mutant of the clinical isolate. Means ± standard deviations from four independent experiments are shown. NS, P>0.05.

In our previous study, we transformed *S. aureus* strains carrying no *psm-mec* with *psm-mec* and investigated the function of *psm-mec*. Use of this method to evaluate gain of function cannot, however, establish the requirement of *psm-mec* to suppress HA-MRSA virulence. From 193 HA-MRSA strains carrying intact *psm-mec*, we selected 18 strains that produce the PSM-mec protein and are susceptible to antibiotics, from which *psm-mec* can be deleted by the antibiotics resistance gene. The *psm-mec-*deleted mutants of these 18 strains were constructed (**Fig. S4**), and were examined whether their PSMα production, AgrA expression, and colony spreading ability were increased, or biofilm formation was decreased. In 13 of 18 strains, each *psm-mec-*deleted mutant produced more PSMα3 than the respective parent strain (**Fig. 6A**). In 14 of 18 strains, each *psm-mec-*deleted mutant produced more PSMα1 and Hld than the respective parent strain (**Fig. 6B**). In 14 of 18 strains, each *psm-mec-*deleted mutant expressed more AgrA than the respective parent strain (**Fig. 6C**). In 15 of 18 strains, each *psm-mec-*deleted mutant had a greater colony spreading capacity than the respective parent strain (**Fig. 6D**). In contrast, in 8 of 18 strains, each *psm-mec-*deleted mutant formed less biofilm than the respective parent strain (**Fig. 6E**). Therefore, in most HA-MRSA strains, *psm-mec* is required for the suppression of PSMα production, AgrA expression, and colony spreading, as well as the promotion of biofilm formation.

**Figure 7:**
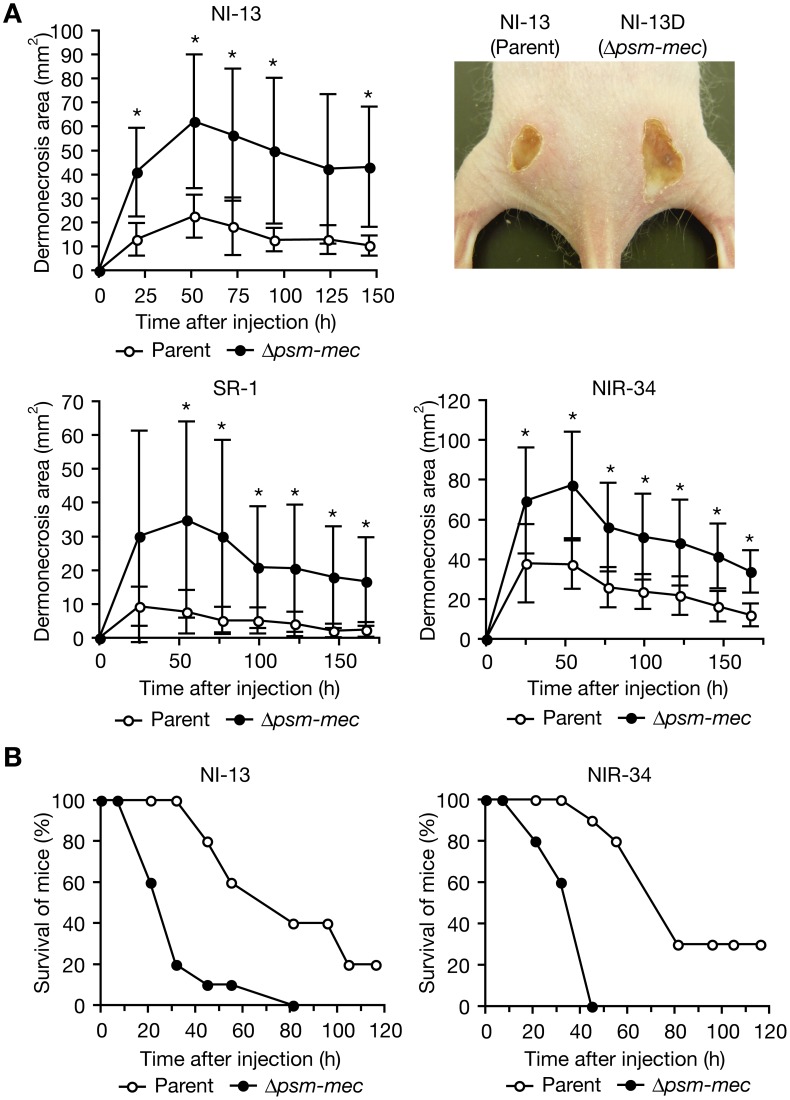
Deletion of *psm-mec* in MRSA clinical isolates increases virulence in mice. (**A**) Mouse skin infection experiments using NI-13, SR-1, NIR-34, and the respective *psm-mec*-deleted mutants were performed. Mice (HR-1, n = 5) were subcutaneously injected with *S. aureus* cells and the dermonecrosis area was measured. Means ± standard deviations from the dermonecrosis areas of five mice are shown. Injected CFUs were as follows; NI-13 and its *psm-mec*-deleted mutant, 4×10^7^ CFU; SR-1 and its *psm-mec*-deleted mutant, 8×10^6^ CFU; NIR-34 and its *psm-mec*-deleted mutant, 2×10^7^ CFU. Black stars indicate that Student's t-test P-values between the parent strain and the *psm-mec*-deleted mutant were less than 0.05. Upper right panel is a representative image of a mouse injected with NI-13 and the *psm-mec*-deleted mutant at 143 h after bacteria injection. (**B**) Mouse systemic infection experiments were performed. ICR mice (n = 10) were intravenously injected with *S. aureus* cells. Injected CFUs were as follows; NI-13 and its *psm-mec*-deleted mutant, 4×10^8^ CFU; NIR-34 and its *psm-mec*-deleted mutant, 4×10^8^ CFU. Log-rank test P-values between the parent strain and the *psm-mec-*deleted mutant in NI-13 and SR-1 are 0.0005 and <0.0001, respectively.

To further verify whether *psm-mec* is needed to suppress HA-MRSA virulence in animals, we examined the virulence of the *psm-mec-*deleted mutants of NI-13, SR-1, and NIR-34, which produced higher amounts of PSMα3 than their respective parent strain, using mouse models of skin infection and systemic infection. In the skin infection model, bacterial virulence was quantitatively evaluated by measuring the dermonecrosis area formed by the *S. aureus* injection [25]. The *psm-mec-*deleted mutants of NI-13, SR-1, and NIR-34 formed a larger area of dermonecrosis than the respective parent strain (**Fig. 7A**). In the mouse systemic infection model, the *psm-mec-*deleted mutants of NI-13 and NIR-34 killed mice faster than the respective parent strain (**Fig. 7B**), although the *psm-mec-*deleted mutant of SR-1 killed mice in the same manner as the parent strain (data not shown). These results suggest that *psm-mec* suppresses the virulence of these HA-MRSA strains against animals.

It was recently revealed that *psm-mec* is located between two regulatory loci, *mecI* and *mecR2*, which are transcribed in the opposite direction of *psm-mec* and regulate the expression of *mecA* [26]. Expression of *mecA* encoding penicillin binding protein 2a interferes with the *agr* system, reduces toxin expression, and attenuates virulence in mice [27], [28]. Deletion of *psm-mec* between *mecI* and *mecR2* might alter the expression of *mecR1-mecI-mecR2* mRNA, resulting in the altered expression of *mecA*. We examined whether *psm-mec*-deleted mutants of 18 HA-MRSA strains alter the expression of *mecA*. In 16 of 18 strains, each *psm-mec-*deleted mutant expressed an amount of *mecA* comparable with that of the respective parent strain (**Fig. S5**). In CR-11 and SR-1 strains, each *psm-mec-*deleted mutant expressed less *mecA* than the respective parent strain (**Fig. S5**). These results indicate that in most HA-MRSA strains, the effect of *psm-mec-*deletion on virulence phenotypes are not related to *mecA* expression, whereas in CR-11 and SR-1 strains, the reduced expression of *mecA* in the *psm-mec-*deleted mutants might contribute to the observed phenotype.

### Concluding remarks

Here, we revealed that *psm-mec* RNA specifically binds *agrA* mRNA encoding an *S. aureus* virulence regulatory factor and inhibits its translation. We further demonstrated that the deletion of *psm-mec* from HA-MRSA strains carrying intact *psm-mec* led to increased expression of AgrA and PSMα. Furthermore, we demonstrated that one-third of HA-MRSA isolates from the Kanto area of Japan harbored -7T>C mutated *psm-mec* or did not carry *psm-mec*. These HA-MRSA strains produced high amounts of PSMα3. These findings support the notion that the mutation or absence of *psm-mec* in HA-MRSA strains leads to the high expression of AgrA, resulting in the high production of exotoxins and high virulence, whereas in almost two-thirds of HA-MRSA strains carrying intact *psm-mec*, the expression of AgrA is inhibited by *psm-mec*, resulting in attenuated virulence. In addition, we demonstrated that the integration of *psm-mec* into the chromosomes of the CA-MRSA strains, which do not carry *psm-mec*, led to a decrease in the expression of AgrA. Thus, we propose that the absence of *psm-mec* is a genetic determinant of the high virulence property of CA-MRSA, i.e., the high expression of *agr* locus. Identification of the *psm-mec* mutation could be a novel method for predicting the virulence properties of MRSA strains. We have revealed that HA-MRSA isolates carrying -7T>C *psm-mec* had closely related genetic backgrounds with isolates carrying intact *psm-mec*. Thus, the -7T>C mutation of *psm-mec* may frequently appear from intact *psm-mec*. In contrast, HA-MRSA isolates carrying no *psm-mec* have genetic backgrounds that differ from those carrying intact or -7T>C *psm-mec*, indicating that isolates not carrying *psm-mec* are evolutionarily distant from isolates carrying intact or -7T>C *psm-mec*. It will be interesting to see whether the ratio of these three MRSA groups in hospitals changes over time in relation to clinical outcome.

AgrA activates the transcription of *psm-mec* [23], [29]. We also confirmed that the expression of *psm-mec* was diminished in the *agr*-null mutant and that *psm-mec* expression was restored by the introduction of *agrBDCA*. This study revealed that *psm-mec* RNA negatively regulates *agrA* translation. These results indicate that AgrA increases the amount of *psm-mec* RNA, *psm-mec* RNA inhibits *agrA* translation, and the decreased amount of AgrA leads to decreased expression of PSMαs as well as of *psm-mec* RNA itself. Thus, the expression of *psm-mec* RNA and AgrA is assumed to be maintained in a steady balance by this feedback loop, and the presence of *psm-mec* might moderately suppress AgrA expression.

Queck *et al*. revealed that *psm-mec* has a positive effect on virulence in mouse skin and systemic infection models of the HA-MRSA strain MSA890, in which the amount of PSM-mec, which has cytolytic activity against human neutrophils, was higher than that of PSMα peptides [29]. Furthermore, the same group revealed that *psm-mec* had no effect on the virulence of HA-MRSA strains Sanger252, BK1406, and BK23684, in which the amount of PSM-mec was not higher than that of PSMα peptides [23], [29]. In the present study, we constructed the *psm-mec-*deleted mutants from 18 clinical isolates of HA-MRSA and demonstrated that *psm-mec* represses the expression of PSMα and AgrA in most of these strains. In addition, we revealed that *psm-mec* suppresses virulence in mouse skin and systemic infection models of at least two HA-MRSA strains. To examine whether the discrepancy between our results and the results by Queck *et al.* was due to differences in the experimental procedure, we examined the virulence of MSA890, Sanger252, and their *psm-mec-*deleted mutants in a mouse systemic infection model. We found that the *psm-mec*-deleted mutant of the MSA890 strain showed decreased virulence compared with the parent strain, whereas the *psm-mec*-deleted mutant of Sanger252 did not show decreased virulence compared with the parent strain (**Fig. S6**), consistent with the reports by Queck *et al.* Therefore, the genetic backgrounds of the HA-MRSA strains, not differences in the assay system, might explain the discrepancy between our results and the results reported by Queck *et al.*

RNAIII was the first identified regulatory RNA in *S. aureus* [9] and regulates the expression of various genes [7]. RNAIII inhibits the translation of *spa* encoding protein A [20], *coa* encoding staphylocoagulase [21], and *rot* encoding a transcription factor [30]. RNAIII carries hairpin loops with a C-rich motif that binds the G-rich sequence of the SD region of target mRNA and inhibits its translation [22], [30]. RNAIII forms an imperfect duplex with target mRNA, which is digested by RNase III, and decreases the stability of target mRNA [20], [31]. RNAIII is stable with a half-life of over 20 min [20], [30]. In the present study, we predicted that the C-rich motif (ACCC) of *agrA* mRNA binds the SD region (GGGU) of *psm-mec* RNA (**Fig. 3A**). We revealed that *psm-mec* RNA slightly destabilizes *agrA* mRNA in an RNase III-dependent manner (**Fig. 4A**). The *psm-mec* RNA was stable with a half-life of approximately 20 min (**Fig. 4D**). Therefore, the molecular mechanism underlying the interaction between *psm-mec* RNA and *agrA* mRNA is similar to that of RNAIII. Because the half-life of *psm-mec* RNA was not changed by the presence of *agrA* mRNA (**Fig. 4D**), it is possible that anhydrotetracycline induced excess amounts of *psm-mec* RNA relative to the amount of *agrA* mRNA, and thus digestion of the *psm-mec* RNA/*agrA* mRNA duplex structure did not affect the half-life of the *psm-mec* RNA. Furthermore, *psm-mec* inhibited the expression of AgrA in an RNase III-independent manner (**Fig. 4C**). These findings suggest that the destabilization of *agrA* mRNA by *psm-mec* RNA and RNase III occurs after the inhibition of *agrA* translation by *psm-mec* RNA. This is consistent with previous reports that translational repression by a small RNA does not require mRNA destabilization in *E. coli* and *S. aureus* [32], [33]. Most small RNAs bind the SD sequence of target RNA, although recent reports indicate that some regulatory RNAs bind the coding region of target mRNA [34], [35]. A unique feature of the interaction between *psm-mec* RNA and *agrA* mRNA is that both RNAs encode proteins and duplex formation involves the coding sequence of both RNAs, i.e., mRNA-mRNA interaction. Because *psm-mec* RNA binds the coding region of *agrA* mRNA, which is far from the ribosome binding site, it is possible that the conformational change of *agrA* mRNA by *psm-mec* RNA prevents ribosome recruitment or the RNA-RNA pairing inhibits translation elongation. Further studies are needed to clarify whether the interaction between *psm-mec* RNA and *agrA* mRNA leads to conformational alteration of the SD structure and to examine the effects of *psm-mec* RNA on the translation initiation and elongation of *agrA* mRNA. An mRNA-mRNA interaction might have a broad role in the regulation of gene expression and should be investigated further.

## Materials and Methods

### Ethics statement

This study was performed in strict accordance with the recommendation of the Fundamental Guidelines for Proper Conduct of Animal Experiment and Related Activities in Academic Research Institutions under the jurisdiction of the Ministry of Education, Culture, Sports, Science, and Technology in Japan, 2006. All mouse protocols followed the Regulations for Animal Care and Use of the University of Tokyo and were approved by the Animal Use Committee at the Graduate School of Pharmaceutical Science at the University of Tokyo (approval number: 19–28). All clinical isolates of MRSA were obtained in accordance with the protocols approved by the ethics committee of Nippon Medical School Hospital (approval number: 18-03-49). All patients provided informed consent prior to donating *S. aureus* isolates. All clinical isolates of MRSA were anonymized because clinical data were not used.

### Bacterial isolates

We collected 325 clinical isolates of MRSA strains from Nippon Medical School Hospital (Bunkyo, Tokyo, Japan), Nippon Medical School Chiba Hokusoh Hospital (Inzei, Chiba, Japan), and Sekino Clinical Pharmacology Clinic (Toshima, Tokyo, Japan) from 2008-2010. These strains were streaked on mannitol sodium chloride plates (Eiken Chemical Inc., Tokyo, Japan) and their utilization of mannitol and high-salt resistance were confirmed. Their minimum inhibitory concentration values against oxacillin were examined and resistance to oxacillin was also confirmed. Bacterial strains used in this study are shown in Table 4.

**Table 4:** Bacterial strains and plasmids used.

Strain or plasmid	Genotypes or characteristics^a^	Source or reference
**Strains**		
*S. aureus*		
RN4220	NCTC8325-4, restriction mutant	[15]
Newman	Laboratory strain, High level of clumping factor	[51]
MW2	CA-MRSA (USA400)	[52]
FRP3757	CA-MRSA (USA300)	[53]
NI strains	40 clinical MRSA isolates from Nippon Medical School Hospital	[13]
NIR strains	126 clinical MRSA isolates from Nippon Medical School Hospital	This study
CR strains	52 clinical MRSA isolates from Nippon Medical School Chiba Hokusoh Hospital	This study
SR strains	107 clinical MRSA isolates from hospital Sekino Clinical Pharmacology Clinic	This study
MN1844	Newman Δ*agr*::*tetM* (transduction from RN6911)	[54]
MN1076	Newman Δ*rnc*::pT1076	This study
MSA890	MRSA strain carrying type-II SCC*mec*	[29]
MSA890Δ*psm-mec*	MSA890 Δ*psm-mec* without an antibiotic resistance marker	[29]
Sanger252	MRSA strain carrying type-II SCC*mec*	[29]
Sanger252Δ*psm-mec*	Sanger252 Δ*psm-mec* without an antibiotic resistance marker	[29]
*E. coli*		
JM109	General purpose host strain for cloning	Takara Bio
BL21(DE3)pLysS	General purpose host strain for expression of recombinant proteins	Takara Bio
**Plasmids**		
pET-9a	T7 promoter based expression vector, Amp^r^	Novagen
pET-9a-agrAHis	pET-9a with His-tagged *agrA*	This study
pKOR3a	Vector for allelic replacement in *S. aureus*, Cm^r^	[55]
pKOR3a-psm-mecT	pKOR3a with psm-mec-cassette; Cm^r^, Tet^r^	This study
pKOR3a-psm-mecP	pKOR3a with psm-mec-cassette; Cm^r^, Phleo^r^	This study
pKOR3a-psm-mec-I	pKOR3a with psm-mec-I-cassette; Cm^r^, Kan^r^	This study
pND50	*E. coli*-*S. aureus* shuttle vector; Cm^r^	[56]
pF	pND50 with intact *psm-mec* from N315	[13]
pM1	pND50 with promoter deficient *psm-mec* (-7T>C)	[13]
pC1	pND50 with F3 Stop *psm-mec*	[12]
pFP	pND50 with codon-replaced *psm-mec*	[12]
p-psm-mec-D	pND50 with partial-deleted *psm-mec*	This study
p-psm-mec-M	pND50 with nucleotides-substituted *psm-mec*	This study
pGP-luc	pND50 with *recF* promoter, −20–27 of *agrA*, and *luc*	This study
pGP-agrA-luc	pND50 with *recF* promoter, −20–717 of *agrA*, and *luc*	This study
pGP-agrA1-luc	pND50 with *recF* promoter, −20–267 of *agrA*, and *luc*	This study
pGP-agrA2-luc	pND50 with *recF* promoter, −20–198 of *agrA*, and *luc*	This study
pCK20	*S. aureus* suicide vector; Cm^r^	[57]
pInt	pCK20 with partial genomic region from RN4220 that can integrate into *S. aureus* chromosome	[13]
pIntF	pInt with intact *psm-mec* from N315	[13]
pW	pInt with *agr* region from Newman	[39]
pMNS	*E. coli*-*S. aureus* shuttle vector carrying Pspac; Amp^r^, Spc^r^	This study
pMNS-agrBDCA	pMNS with *agrBDCA* from Newman	This study
pMutinT3	*S. aureus* suicide vector; Erm^r^	[36]
pT1076	pMutinT3 with partial *rnc* from NCTC8325-4	This study
pIntE	pMutinT3 with partial genomic region from RN4220 that can integrate into *S. aureus* chromosome	This study
pIntE-F	pIntE with intact *psm-mec* from N315	This study
pD2	pND50 with D2-mutated *psm-mec*	This study
pD3	pND50 with D3-mutated *psm-mec*	This study
pD4	pND50 with D4-mutated *psm-mec*	This study
pD5	pND50 with D5-mutated *psm-mec*	This study
pNDX1	pND50-based *S. aureus*-*E. coli* shuttle vector carrying TetR and *xyl/tet* from pWH353; Cm^r^	[58]
pNDX1-F	pNDX1 with intact *psm-mec* from N315	[55]
pKE516	*S. aureus*-*E. coli* shuttle vector, Erm^r^, Amp^r^	[38]
pKE516-F	pKE516 with intact *psm-mec* from N315	This study

a Amp, ampicillin; Cm, chloramphenicol; Tet, tetracycline; Phleo, phleomycin; Kan, kanamycin; Spc, spectinomycin.

### DNA manipulation

To regulate the expression of AgrA protein under the control of IPTG, pMNS was constructed by fusing pMutinT3 [36] and pTetON [37]. pMNS contains the transcription terminators, Pspac promoter, *lacZ*, and *lacI* from pMutinT3 and pE194 *ori*, pUC *ori*, and the spectinomycin resistance gene from pTetON. pMNS is compatible with pKE516 [38]. Plasmids used in this study are shown in Table 4.

### Preparation of a polyclonal antibody against AgrA

A DNA fragment containing the *agrA* gene was amplified by polymerase chain reaction (PCR) using oligonucleotide primer pairs agrA-HisC-F and agrA-HisC-R and pW as a template [39]. The amplified DNA fragment was phosphorylated by T4 polynucleotide kinase and self-ligated, resulting in pW-agrAHis. The DNA fragment was amplified by PCR using oligonucleotide primer pairs AgrA-F-NdeI and AgrA-R-BamHI and pW-agrAHis as a template. The amplified DNA fragment was inserted into pET-9a at the *Nde* I and *BamH* I sites, resulting in pET-9a-agrAHis. *E. coli* BL21 (DE3) carrying pLysS was transformed with pET-9a-agrAHis. Transformants were cultured in Terrific broth containing 1 M sorbitol and 10 mM betaine at 25°C according to Koenig RL *et al.* [8]. Isopropyl β-D-1-thiogalactopyranoside (0.4 mM) was added to the culture at A_600_ = 0.3 and cultured further for 2.5 h. Cells were collected and lysed by freezing and thawing, and subsequent sonication in a lysis buffer (20 mM Tris-HCl [pH 7.9], 6 M guanidine hydrochloride, 0.5 M NaCl). His-tagged AgrA was purified using a Ni column (ProBond Resin, Life Technologies, Tokyo, Japan) according to the manufacturer's protocol. His-tagged AgrA (0.5 mg) was subcutaneously injected into a Japanese white rabbit 5 times at 2-week intervals. Blood was collected from the rabbit and used for IgG purification by protein G-Sepharose.

### Western blotting for AgrA

*S. aureus* overnight culture was inoculated into a 100-fold amount of fresh tryptic soy broth (TSB) and cultured for 24 h or 15 h at 37°C. *S. aureus* cells were collected by centrifugation from 650 µl of culture and suspended in a lysis buffer (10 mM Tris-HCl [pH 8.0], 1 mM EDTA [pH 8.0], 25 µg/ml lysostaphin) and incubated at 37°C for 30 min. The sample was sonicated and centrifuged at 10,000 *g* for 10 min. The amount of protein in the supernatant was measured by the Bradford method and the protein concentration of different samples was equalized by adding a buffer. Proteins were electrophoresed in 15% sodium dodecyl sulfate (SDS)-polyacrylamide gel at 100 V for 3 h. Proteins were transferred from the gel to a membrane (Immobilon-P, Millipore) in buffer (10 mM CAPS, 20% methanol) at 150 mA for 3 h. The membrane was treated with a blocking buffer (Tris-buffered saline with Tween 20 [TBST] containing 5% Easy Blocker [GeneTex, Irvine, CA]) at room temperature for 1 h. The membrane was treated with a blocking buffer containing 1∶1000 anti-AgrA IgG at room temperature for 1 h. After washing with TBST, the membrane was treated with a blocking buffer containing 1∶2000 anti-rabbit IgG conjugated with alkaline phosphatase at room temperature for 1 h. After washing with TBST, the membrane was treated with a staining buffer (100 mM Tris-HCl [pH9.5], 100 mM NaCl, 50 mM MgCl_2_, 2% nitro-blue tetrazolium/5-bromo-4-chloro-3′-indolyphosphate) for 5 min. The band intensity was measured by densitometry scanning (Image J 1.45 s, NIH).

### Gel shift assay

DNA fragments encoding *psm-mec* mRNA or *agrA* mRNA were amplified by PCR using the primers listed in Table S2 and pF and pGP-agrA-luc as a template, and used as templates for *in vitro* transcription. *In vitro* transcription was performed using the T7 RiboMAX Express Large Scale RNA Production System (Promega). The 5′ end-labeling of dephosphorylated RNA was performed with T4 polynucleotide kinase and [γ-^32^P]ATP. Gel shift assays were performed with 0.13 pmol of labeled RNA (final 19 nM) and various doses of nonlabeled RNA in 7 µl of binding buffer (10 mM Tris-HCl, pH8.0, 30 mM KCl) using our protocol modified from Kawamoto *et al.* and Antal *et al.* [40], [41]. The samples were incubated at 95°C for 1 min and at 37°C for 90 min. Two microliters of 50% glycerol were added to the samples, which were then electrophoresed in a 6% polyacrylamide gel in 45 mM Tris-borate at 4°C. The gels were dried and RNA-RNA interactions were analyzed by phosphoimaging using Typhoon (GE) and Image Gauge v. 4.23 software (Fujifilm).

### Mouse model

The mouse skin infection experiment was performed according to Bunce *et al.* [25]. Female 6-week old Hos:HR-1 mice were purchased from Hoshino Laboratory Animals (Ibaraki, Japan). *S. aureus* overnight culture was inoculated into 100-fold amounts of fresh TSB and cultured to A_600_ = 0.5. Cells were centrifuged and suspended in phosphate-buffered saline (PBS). Colony forming units (CFU) were measured by spreading the suspended cells on TSB agar plates. Mice were anesthetized with pentobarbital and subcutaneously injected with the suspended bacterial cells containing 5% microbeads (Cytodex 1, GE Healthcare). The inflamed area around the injection site was measured daily (length [L] x width [W]). For mouse systemic infection, *S. aureus* overnight culture was inoculated into 100-fold amounts of fresh TSB and cultured for 20 h. *S. aureus* cultures were centrifuged and cells were suspended in PBS. Bacterial suspension (100 µl) was injected into the tail vein of 7-week-old female ICR mice. Survival after injection was monitored. All mice were killed after the experiment.

### Protein separation by 2-DE

Overnight cultures of *S. aureus* strains were inoculated into 100-fold amounts of fresh TSB containing 12.5 µg chloramphenicol/ml and cultured for 14 h at 37°C. One milliliter of culture was centrifuged at 10,000 *g* for 5 min at 4°C and the pellet was frozen in liquid nitrogen. Pellets were resolved with 360 µl of PBS containing 4.8 U DNase, 9.6 µg RNase A, and 9.6 µg lysostaphin, and incubated for 60 min at room temperature. Cell lysates were centrifuged at 10,000 *g* for 5 min at 4°C. TCA was added to the samples (final 10%) and the samples were incubated for 60 min at 4°C. The lysates were centrifuged at 10,000 *g* for 60 min at 4°C, and the precipitates were washed with ethanol twice, and resolved with sample buffer (7 M urea, 2 M thiourea, 50 mM DTT, 4% CHAPS, 0.2% bio-lyte 3/10 ampholyte [Bio-rad], 0.001% BPB). The first isoelectric focusing was performed using 11 cm pH 4–7 IPG strips (Bio-rad) and the PROTEAN IEF system (Bio-rad). The samples (60 µg protein/200 µl sample buffer) were applied to an IPG strip rehydrated for 12 h, and isoelectrically focused at 250 V for 15 min, 8000 V for 6 h, and 500 V for 24 h. Each IPG gel strip was equilibrated in buffer (375 mM Tris-HCl (pH 8.8), 6 M urea, 20% glycerol, 2% SDS, 130 mM DTT) for 20 min and in 2.5% iodoacetamide for 10 min. The IPG gel strips were embedded onto 12.5%-SDS polyacrylamide gel (16 cm×16 cm) using 1% low-melting agarose. The second dimension electrophoresis was performed at a constant 200 V for 3 h at 4°C. Gels were subsequently stained with Coomassie Brilliant Blue.

### Construction of the *psm-mec* integrated CA-MRSA strains

DNA fragments containing the kanamycin resistance gene, *psm-mec*, and upstream and downstream genomic regions of the desired integration site were spliced together by overlap extension PCR. The *psm-mec*-I-cassette was inserted into the *Sma* I site of pKOR3a, resulting in pKOR3a-psm-mec-I (**Fig. S1A**). MW2 and FRP3757 strains were transformed with the plasmid and the *psm-mec-*integrated mutants were obtained using the previous method [12]. The desired integration by double recombination event was confirmed by Southern blot analysis (**Fig. S1B**). We confirmed that the 5′ and 3′ ends of *psm-mec* RNA transcribed from the genome-integrated *psm-mec* were the same as those transcribed from plasmid-encoded *psm-mec* (pF) by a circularized RACE experiment [42].

### Determination of the 3′-terminus of *psm-mec* mRNA by S1 mapping

The DNA fragment containing *psm-mec* was amplified by PCR using primers S2 and F5, and pF as a template. The DNA fragment was digested with *Nde* I and its 3′-teminus was labeled with [α-^32^P]-dATP using a Klenow fragment. The labeled DNA fragment was electrophoresed in 5% native polyacrylamide gel in 0.5 x TBE. A single strand (242 bases) of the ^32^P-labeled DNA was cut out from the gel by UV-shadowing and purified. Maxam-Gilbert sequencing ladders were obtained from the end-labeled probe DNA [43]. The end-labeled probe DNA and total RNA of Newman transformed with *psm-mec* were incubated at 75°C for 10 min and 37°C for 2 h in a buffer (20 mM HEPES-KOH [pH 6.5], 80% formamide, 400 mM NaCl). The polynucleotides in the sample were digested with S1 nuclease in a buffer (30 mM sodium acetate [pH 4.6], 0.3 M NaCl, 1 mM ZnSO_4_) at 37°C for 15 min. The reaction was terminated by adding PCI (phenol: chloroform: isoamylalcohol = 25∶24∶1). S1-reacted products and Maxam-Gilbert sequencing ladders were electrophoresed in 8 M urea-7.5% polyacrylamide gel. The gels were dried and DNA was analyzed by phosphoimaging using Typhoon (GE).

### Reporter assay

DNA fragments containing the *recF* promoter region and the *agrA* region (−20–717) were amplified by PCR and inserted into pluc [44], resulting in a pluc-recFP-agrA. The *agrA* ORF and *luc* ORF were fused by PCR using primers agrA-R and lucATG-F, resulting in pGP-agrA-luc. Deletion mutants of *agrA* region were constructed by PCR using pGP-agrA-luc as a template and primers listed in Table S2, resulting in pGP-luc, pGP-agrA1-luc, pGP-agrA2-luc. The DNA fragment containing *psm-mec* was inserted into these plasmids, resulting in +F constructs. Constructs of all plasmids were confirmed by sequencing. *S. aureus* RN4220 strain was transformed with the plasmids. The plasmids were transferred to Newman strain using phage 80α. Overnight cultures of the Newman strains transformed with the plasmids were inoculated into 100-fold amounts of fresh TSB, and cultured to A_600_ = 1. Cells were collected by centrifugation at 10,000 *g* for 1 min. Cells were lysed and luciferase activity was measured according to Hanada *et al.* [45].

### Determination of RNA half-life

The half-life of mRNA was determined as previously described with slight modification [46]. Overnight cultures of *S. aureus* were inoculated into 100-fold amounts of fresh TSB and cultured to A_600_ = 3. After treatment with rifampicin (300 µg/ml), samples were collected at the indicated time-points and immediately treated with RNAprotect Bacteria Reagent (Qiagen). Total RNA was extracted using an RNeasy mini kit (Qiagen) according to the manufacturer's protocol. RNA was electrophoresed onto a 1% agarose gel containing 6.6 M formaldehyde and transferred to a nylon membrane (GeneScreen Plus, Perkin Elmer). A DNA probe of the *agrA* gene or the *psm-mec* gene was labeled with [^32^P]-dCTP by random priming. Hybridization was performed at 42°C. Band intensity was measured by densitometry scanning (Image J 1.45 s, NIH). The amount of the detected RNA was normalized to the amount of 16S rRNA and the time-point at which the amount of RNA reached half that at time 0 min was calculated by exponential approximation.

### Sequencing of *psm-mec* of clinical isolates

DNA fragments containing *psm-mec* were amplified by PCR using genomic DNAs of clinical isolates as a template and primer pairs of S2 and S3. The nucleotide sequence was determined using primers S2 and S3 (**Table S2**). DNA fragments containing intact *psm-mec* and mutated *psm-mec* were amplified by PCR using primer pairs of S2-XbaI and S3-SacI, and inserted into the *Xba* I and *Sac* I sites of pND50, resulting in pF, pM1, pD2, pD3, pD4, and pD5. The effects of these plasmids on the Newman strain were evaluated (**Fig. S3**).

### *spa* typing

Typing of the polymorphic region of the protein A gene (*spa*) was performed as described previously [47]. Purified *spa* PCR products were sequenced, and short-sequence repeats were assigned using the *spa* database website (http://tools.egenomics.com./Public/Login.aspx).

### SCC*mec* typing

Multiplex PCRs were performed to identify the SCC*mec* types according to the established method [48]. Primer sets M-PCR1 and M-PCR2 were used. When DNA was not amplified by using one of the primer set, the stain was classified as non-typed.

### Construction of the *psm-mec*-deleted MRSA strains and the *rnc-*deleted Newman strain

DNA fragments containing antibiotic-resistant gene and the upstream and downstream regions of *psm-mec* were spliced together by overlap extension PCR, resulting in a psm-mec-cassette. The psm-mec-cassette was inserted into the *Sma* I site of pKOR3a, resulting in pKOR3a-psm-mec. MRSA strains were transformed with the plasmid and the *psm-mec*-deleted mutants were obtained using the previously reported method [12]. The desired deletion of *psm-mec* by double homologous recombination was confirmed by Southern blot analysis (**Fig. S4**). To disrupt *rnc* in the Newman strain, a single-cross over recombination method was used, as reported previously [49].

### Measurement of PSMs

The amount of PSM was measured as previously described with slight modification [12]. Overnight bacterial cultures (50 µl) were inoculated into 5 ml fresh tryptic soy broth and aerobically cultured at 37°C for 15 h without antibiotics. The cultures were centrifuged and the supernatants were evaporated using a centrifuge evaporator (CC-105, TOMY, Tokyo, Japan). The evaporated products were solved in 40% acetonitrile and centrifuged at 20,400 *g* for 5 min. The supernatants were evaporated using a centrifuge evaporator, and the evaporated products were dissolved in water, and analyzed by reversed phase-high performance liquid chromatography. Chromatography was performed using a SOURCE 5RPC ST 4.6/150 column (GE Healthcare, Tokyo, Japan) and 50% acetonitrile in 0.1% trifluoroacetic acid for 3 min and a water/acetonitrile gradient in 0.1% trifluoroacetic acid from 50 to 90% acetonitrile for 20 min at a flow rate of 1 ml/min (600E, Waters, Milford, MA). Absorbance at 215 nm was detected using a 2998 Photodiode Array Detector (Waters). The molecular mass in the respective peak was determined using liquid chromatography-electrospray ionization mass spectrometry (LC 1100 series, Agilent Technologies, Santa Clara, CA; ESI-MS, Bio-TOFQ, Bruker Daltonics, Billerica, MA) and the respective PSMs were identified as previously described [12]. Hld and PSMα1 were not separated in this system.

### Colony spreading assay

The colony spreading assay was basically performed according to our previous method [50]. Two microliters of *S. aureus* overnight culture were spotted onto soft TSB agar plates containing 0.24% agar, and was incubated for 24 h at 37°C. The diameter of the giant colony was measured.

### Biofilm formation assay

The biofilm formation assay was basically performed according to our previous method [12]. *S. aureus* overnight culture was inoculated into a 200-fold amount of fresh TSB containing 0.25% glucose in 96-well polystyrene plates and cultured for 3 days at 37°C. Cells attached to the plate were stained with safranin and measured by A_490_.

## Supporting Information

Figure S1 Integration of *psm-mec* into chromosomes of CA-MRSA strains. (**A**) The genomic region that was integrated with *psm-mec* is schematically represented as the *psm-mec-*integrated CA-MRSA strain. Probe DNA regions, construct of targeting plasmid, genomic region of CA-MRSA (type-IV SCC*mec*) are presented above. Predicted lengths of DNA fragments that were digested with *Sph* I are presented. (**B**) Genomic DNAs of MW2, FRP3757, and their *psm-mec*-integrated mutants were digested with *Sph* I and subjected to Southern blot analysis using probes 1 and 2. P indicates the parent strain. A, B, and C means independently obtained *psm-mec* integrated mutants.
(TIF)

Figure S2 Determination of 3′-terminus of *psm-mec* RNA. The 3′-terminus of *psm-mec* RNA was determined by S1 mapping. S1-digested products and Maxam-Gilbert sequencing ladders were electrophoresed in 8 M urea-7.5% polyacrylamide gel. Lanes 1 and 2 represent products that were digested with 75 U and 450 U of S1 nuclease, respectively. Lanes AG, CT, and C represent Maxam-Gilbert sequencing ladders. The nucleotide sequences of *psm-mec* RNA and the antisense RNA are presented on the left side of the panel. Black stars represent the 3′-terminus of *psm-mec* RNA determined by the migration of the S1-digested product.
(TIF)

Figure S3 Effect of the mutated *psm-mec* sequences found in clinical MRSA isolates on *S. aureus* Newman strain. (**A**) The amount of PSM-mec in the culture supernatant of the Newman strain transformed with empty vector (pND50), intact *psm-mec* (pF), D1-mutated *psm-mec* (pM1), D2-mutated *psm-mec* (pD2), D3-mutated *psm-mec* (pD3), D4-mutated *psm-mec* (pD4), or D5-mutated *psm-mec* (pD5) was measured. The vertical axis represents the relative amount of PSM-mec against that of Newman transformed with pF. Means ± standard deviations from four independent experiments are shown. Student t-test P-values between pF-transformed Newman and other strains are presented. NS, P>0.05. ND, not detected. (**B**, **C**) The amount of PSMα3 (B) and PSMα1+Hld (C) of the *psm-mec*-transformed strains described above was measured. The vertical axis represents the relative amount of PSMα3 against that of Newman transformed with pND50. Means ± standard deviations from four independent experiments are shown. Student t-test P-values between pF-transformed Newman and other strains are presented. NS, P>0.05. (**D**) Colony spreading ability of the above *psm-mec-*transformed strains was evaluated. Two microliters of *S. aureus* overnight cultures was spotted onto soft agar plates and incubated at 37°C for 8 h. Diameter of the giant colony was measured. Means ± standard deviations from four independent experiments are shown. Student t-test P-values between pF-transformed Newman and other strains are presented. NS, P>0.05. (**E**) Biofilm formation of the above *psm-mec-*transformed strains was evaluated. *S. aureus* was cultured in polystyrene plates for 3 days and the biofilm was stained by safranin. Means ± standard deviations from six independent experiments are shown. Student t-test P-values between pF-transformed Newman and other strains are presented. NS, P>0.05.
(TIF)

Figure S4 Deletion of *psm-mec* from clinical MRSA isolates. (**A**) Schematic representation of the genomic region around *psm-mec* in type-II SCC*mec*. *psm-mec* was deleted by *tetL* in NI-13, NI-18, CR-11, CR-12, CR-18, CR-29, CR-38, SR-8, NIR-34, NIR-45, and NI-7 strains. *psm-mec* was deleted by the phleomycin resistance gene in NI-4, NI-22, NI-36, SR-1, NIR-121, NI-3, and NI-38 strains. DNA fragment lengths that were digested by *Bgl* II are presented. (**B**) Genomic DNAs of 18 clinical MRSA strains and their *psm-mec*-deleted mutants were digested with *Bgl* II and subjected to Southern blot analysis using the probes presented in (A). P indicates the parent clinical strain. A, B, and C indicate independently obtained *psm-mec*-deleted mutants.
(TIF)

Figure S5 Expression of *mecA* in the *psm-mec-*deleted mutants of clinical isolates. Northern blot analysis was performed to detect *mecA* mRNA in the *psm-mec-*deleted mutants and clinical isolates. Total RNA was extracted from cultures at the log phase (A_600_ = 0.5) and electrophoresed. rRNA stained with ethidium bromide is shown. Data are representative from three independent experiments.
(TIF)

Figure S6 Virulence of the *psm-mec*-deleted mutants of MSA890 and Sanger252 in a mouse systemic infection model. ICR mice (n = 10) were intravenously injected with *S. aureus* cells. Injected CFUs were as follows: MSA890 and its *psm-mec*-deleted mutant, 2×10^8^ CFU; Sanger252 and its *psm-mec*-deleted mutant, 2×10^8^ CFU. Log-rank test P-value between MSA890 and its *psm-mec-*deleted mutant is 0.0489.
(TIF)

Table S1 Identification of proteins upregulated by *psm-mec* RNA in the FRP3757 strain.
(DOC)

Table S2 Primers used in the study.
(DOC)
