# Supplementary figures and images for: Mobile Genetic Element SCCmec-encoded psm-mec RNA Suppresses Translation of agrA and Attenuates MRSA Virulence
Source: PLoS Pathog. 2013 Apr 4;9(4):e1003269. doi: 10.1371/journal.ppat.1003269 (PMC3617227; doi:10.1371/journal.ppat.1003269)

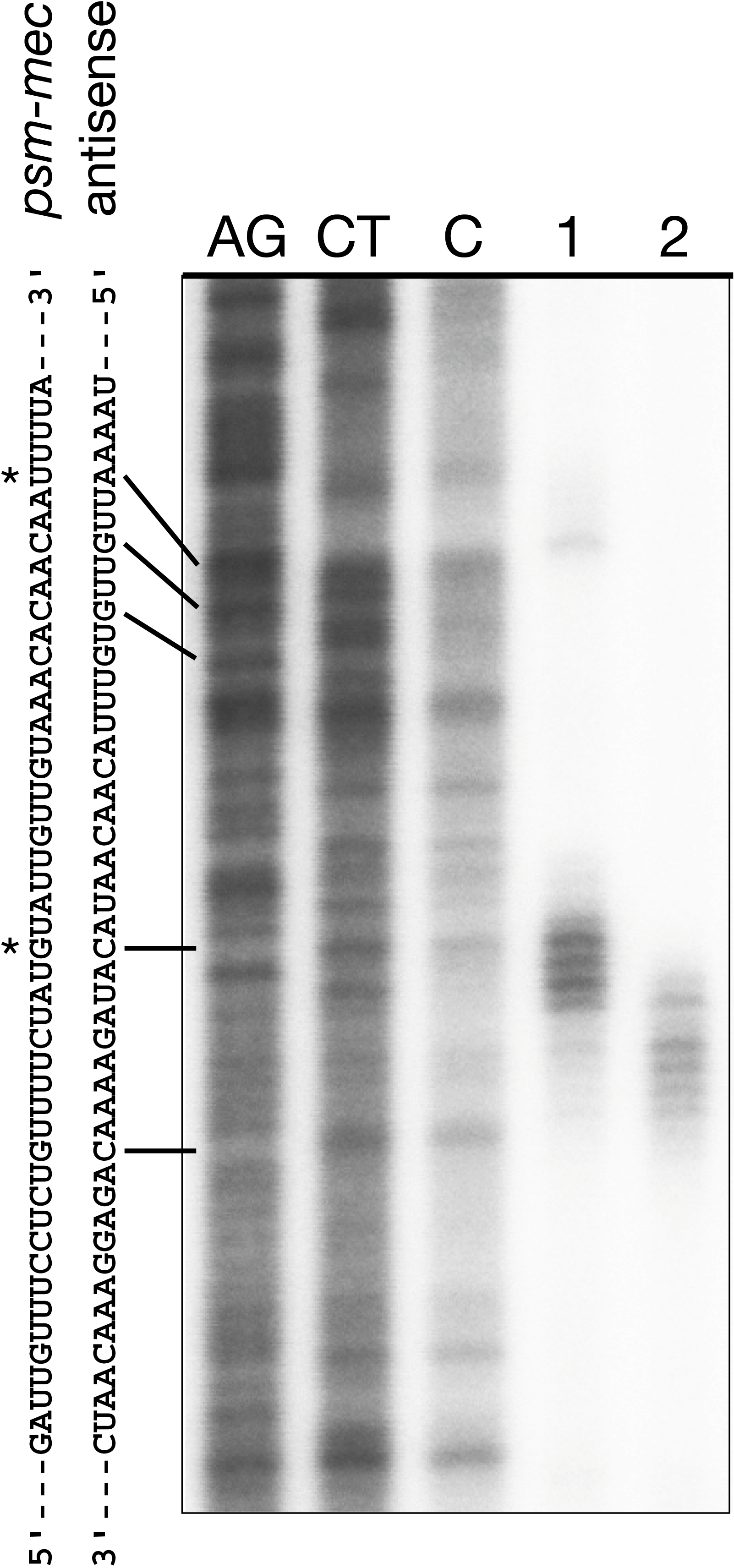

Supplement: Figure S2 — Determination of 3′-terminus of psm-mec RNA. The 3′-terminus of psm-mec RNA was determined by S1 mapping. S1-digested products and Maxam-Gilbert sequencing ladders were electrophoresed in 8 M urea-7.5% polyacrylamide gel. Lanes 1 and 2 represent products that were digested with 75 U and 450 U of S1 nuclease, respectively. Lanes AG, CT, and C represent Maxam-Gilbert sequencing ladders. The nucleotide sequences of psm-mec RNA and the antisense RNA are presented on the left side of the panel. Black stars represent the 3′-terminus of psm-mec RNA determined by the migration of the S1-digested product. (TIF) [file ppat.1003269.s002.tif]

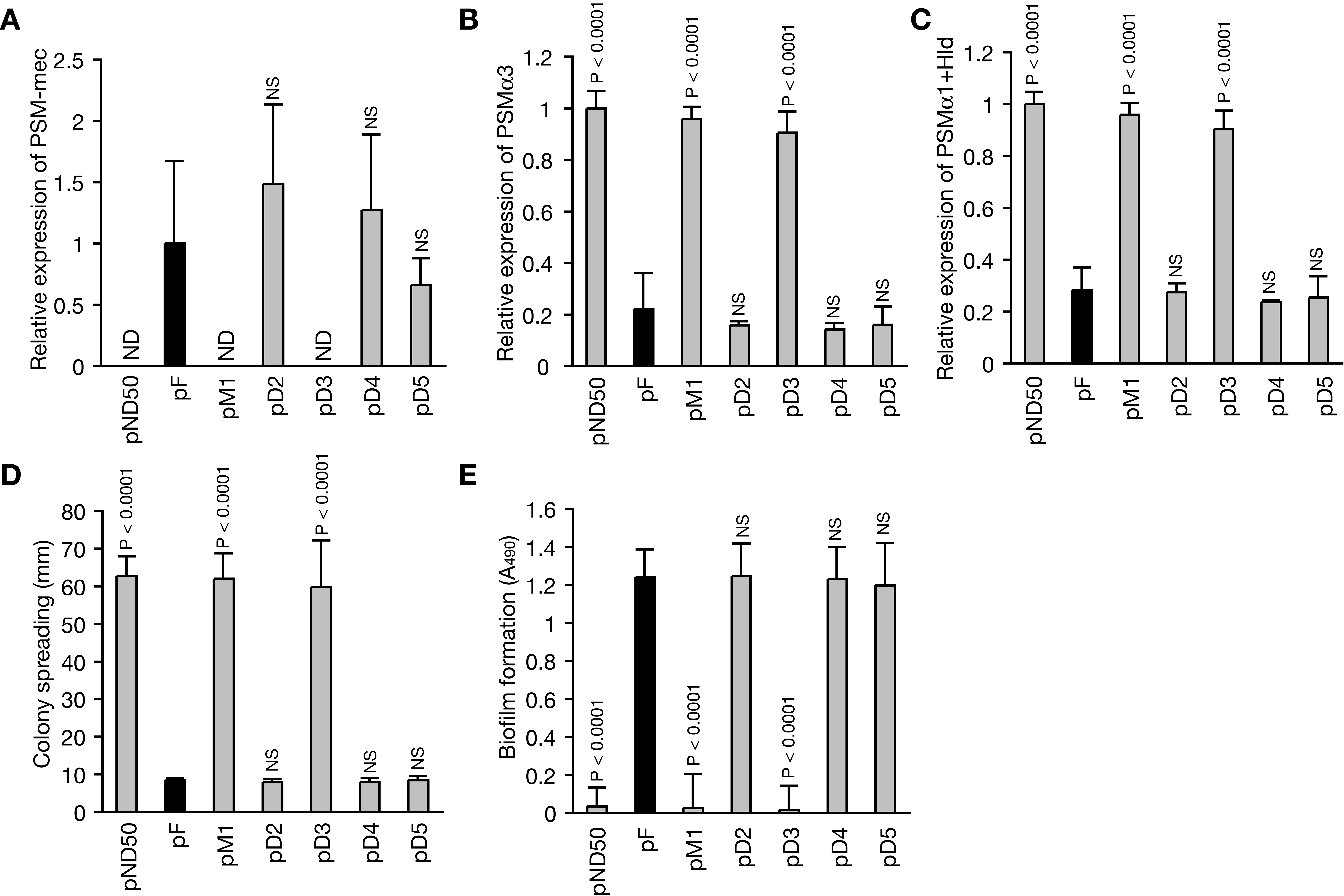

Supplement: Figure S3 — Effect of the mutated psm-mec sequences found in clinical MRSA isolates on S. aureus Newman strain. (A) The amount of PSM-mec in the culture supernatant of the Newman strain transformed with empty vector (pND50), intact psm-mec (pF), D1-mutated psm-mec (pM1), D2-mutated psm-mec (pD2), D3-mutated psm-mec (pD3), D4-mutated psm-mec (pD4), or D5-mutated psm-mec (pD5) was measured. The vertical axis represents the relative amount of PSM-mec against that of Newman transformed with pF. Means ± standard deviations from four independent experiments are shown. Student t-test P-values between pF-transformed Newman and other strains are presented. NS, P>0.05. ND, not detected. (B, C) The amount of PSMα3 (B) and PSMα1+Hld (C) of the psm-mec-transformed strains described above was measured. The vertical axis represents the relative amount of PSMα3 against that of Newman transformed with pND50. Means ± standard deviations from four independent experiments are shown. Student t-test P-values between pF-transformed Newman and other strains are presented. NS, P>0.05. (D) Colony spreading ability of the above psm-mec-transformed strains was evaluated. Two microliters of S. aureus overnight cultures was spotted onto soft agar plates and incubated at 37°C for 8 h. Diameter of the giant colony was measured. Means ± standard deviations from four independent experiments are shown. Student t-test P-values between pF-transformed Newman and other strains are presented. NS, P>0.05. (E) Biofilm formation of the above psm-mec-transformed strains was evaluated. S. aureus was cultured in polystyrene plates for 3 days and the biofilm was stained by safranin. Means ± standard deviations from six independent experiments are shown. Student t-test P-values between pF-transformed Newman and other strains are presented. NS, P>0.05. (TIF) [file ppat.1003269.s003.tif]

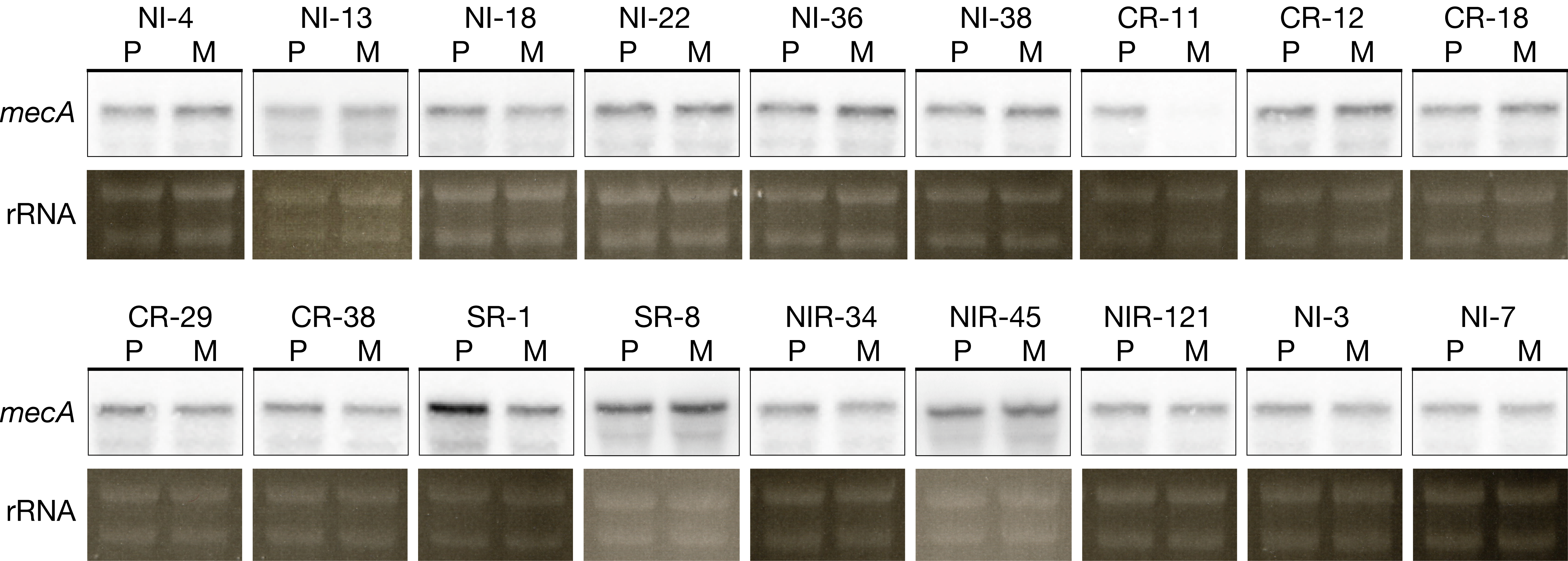

Supplement: Figure S5 — Expression of mecA in the psm-mec-deleted mutants of clinical isolates. Northern blot analysis was performed to detect mecA mRNA in the psm-mec-deleted mutants and clinical isolates. Total RNA was extracted from cultures at the log phase (A600 = 0.5) and electrophoresed. rRNA stained with ethidium bromide is shown. Data are representative from three independent experiments. (TIF) [file ppat.1003269.s005.tif]

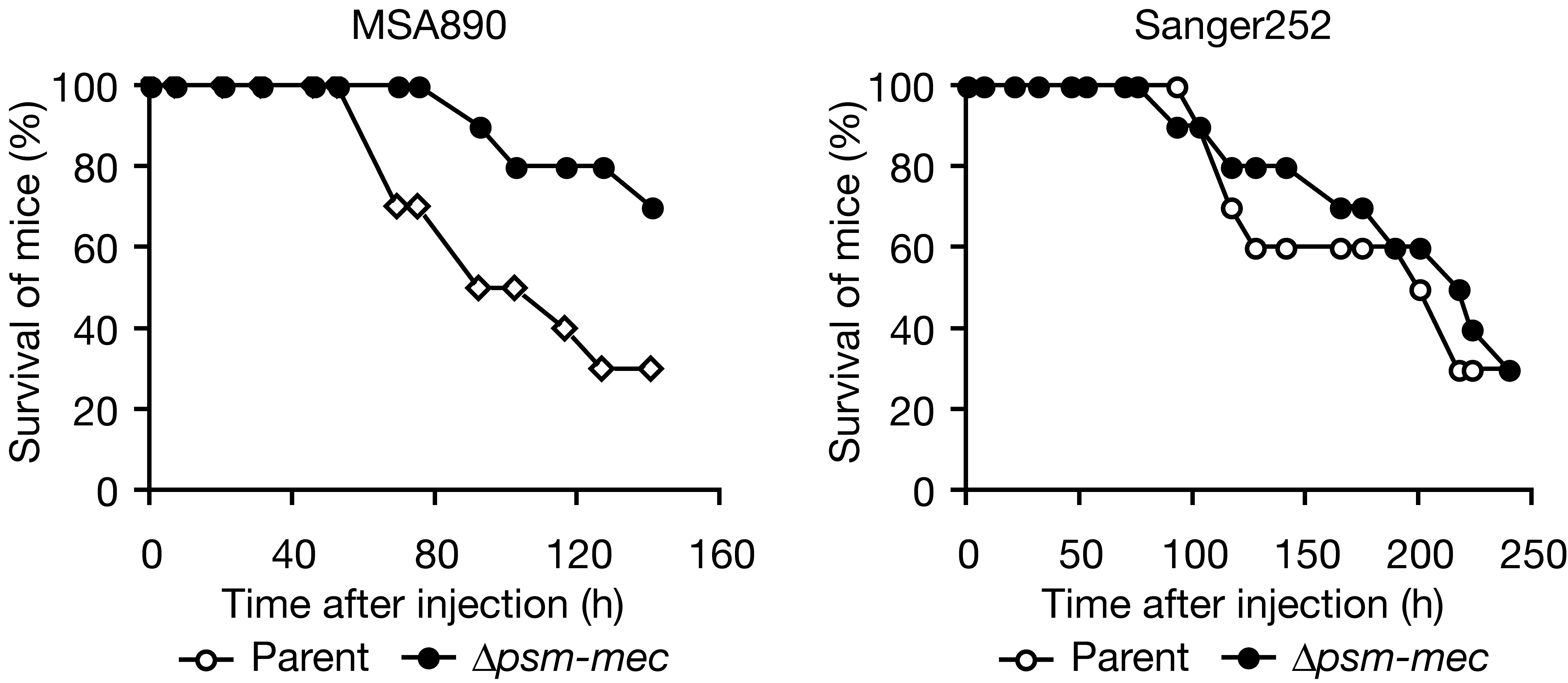

Supplement: Figure S6 — Virulence of the psm-mec-deleted mutants of MSA890 and Sanger252 in a mouse systemic infection model. ICR mice (n = 10) were intravenously injected with S. aureus cells. Injected CFUs were as follows: MSA890 and its psm-mec-deleted mutant, 2×10^8^ CFU; Sanger252 and its psm-mec-deleted mutant, 2×10^8^ CFU. Log-rank test P-value between MSA890 and its psm-mec-deleted mutant is 0.0489. (TIF) [file ppat.1003269.s006.tif]
