# Supplementary material for: Mobile Genetic Element SCCmec-encoded psm-mec RNA Suppresses Translation of agrA and Attenuates MRSA Virulence
Source: PLoS Pathog. 2013 Apr 4;9(4):e1003269. doi: 10.1371/journal.ppat.1003269 (PMC3617227; doi:10.1371/journal.ppat.1003269)
Supplement: Table S1 — Identification of proteins upregulated by psm-mec RNA in the FRP3757 strain. (DOC) [file ppat.1003269.s007.doc]

**Table S1.** Identification of proteins upregulated by *psm-mec* RNA in the FRP3757 strain.

| Isoelectric Point (pI) | | Molecular weight (kDa) | | Protein predicted | Peptides mached | Sequence coverage |
| --- | --- | --- | --- | --- | --- | --- |
| Observed | Theoretical | Observed | Theoretical |  |  | (%) |
| 5.5 | 5.2 | 60 | 60.8 | Urocanate hydratase (HutU)  (gi: 15927911) | 35 | 50 |
| 5.2 | 5.2 | 48 | 48.5 | Protein A (Spa)  (gi: 120864982) | 13 | 34 |
| 5.2 | 5.1 | 38 | 36.7 | D-Lactate dehydrogenase (Ddh)  (gi: 57651032) | 28 | 53 |

The protein band stained with Coomassie Brilliant Blue was excised and digested in-gel with trypsin, desalted with ZipTip C18 (Millipore). Matrix-assisted laser desorption ionization time-of-flight mass spectrometry analysis of the samples was performed using a AXIMA Performance (Shimazu/KRATOS). Database searching was performed using the Mascot search program (www.matrixscience.com).
