# Supplementary material for: Mobile Genetic Element SCCmec-encoded psm-mec RNA Suppresses Translation of agrA and Attenuates MRSA Virulence
Source: PLoS Pathog. 2013 Apr 4;9(4):e1003269. doi: 10.1371/journal.ppat.1003269 (PMC3617227; doi:10.1371/journal.ppat.1003269)
Supplement: Table S2 — Primers used in the study. (DOC) [file ppat.1003269.s008.doc]

**Table S2.** Primers used in the study

| Purpose or target | Primer | Sequence (5'-3') |
| --- | --- | --- |
| In vitro transcription | T7-psmmec-F | TAATACGACTCACTATAGGGATCTTAAATAGAAAGAGGGTATGCAT |
|  | T7-psmmec-R | CATAGAAAACAGAGGAAACAATCAAGTCG |
|  | T7-agrA-F | CGGACTAGTGGAGGCTTACTTGTCTGCTTTC |
| Insertion of *psm-mec* | S3-Km-F | GGGGTAGGCAATTATGGAAAATAAAATAAGCGAACCATTTGAGGTGAT |
|  | Km-R | GGGACCCCTATCTAGCGAAC |
|  | S2 | CAATTCACTTGTCTTAAACTTTGTAGAAAAAGAAG |
|  | S3 | TATTTTATTTTCCATAATTGCCTACCCCATAAG |
|  | preF-F | AAGGCATTCC GACAAATTGA |
|  | S2-preF-R | AGTTTAAGACAAGTGAATTGTTATTGCTGACTTGCCATTAATTCTGC |
|  | postF-F | GTTCGCTAGATAGGGGTCCCTCAGCAATAAAAAAATAAATCATAATGAG |
|  | postF-R | TGGACAACTTAAGCCAGGGTA |
| S1 mapping | F5 | CTTAATGTATCTTAAATAGAAAGAG |
| Reporter-fusions | recF-P-F | GAGGAGCTCAAAAGGTGACGACTCGGTAA |
|  | recF-P-R | GGTGGTACCTCTTGAACCAAAATAATCACTCCA |
|  | agrA-F-KpnI | GGTGGTACCAGCCATAAGGATGTGAATGTATGAAA |
|  | agrA-R-XbaI | TCTTCTAGATATTTTTTTAACGTTTCTCACCGATG |
|  | agrA-R | TATTTTTTTAACGTTTCTCACCGATG |
|  | lucATG-F | ATGGAAGACGCCAAAAACATAAAGATTTGTCTACAAAGTTGC |
|  | agrA27-R | ATCGTCTTCGCAAATGAAAATTTTC |
|  | agrA198-R | CAGTTGAAAGTTGAATATCTAAAAAGTAACAGCCTATGTCATTC |
|  | agrA267-R | ACTCGTAACGAAAATAATGTTACC |
|  | S2-EcoRI | GAAGAATTCAATTCACTTGTCTTAAACTTTGTAGAAAAAGAAG |
|  | S3-SacI | GAGGAGCTCTATTTTATTTTCCATAATTGCCTACCCCATAAG |
| Mutation of *psm-mec* | mecD-F | CATTATTGATTTAATCAAGACTTGCATTCAGGC |
|  | mecD-R | TACCCTCTTTCTATTTAAGATACATTAAGT |
|  | mecW-F | TTACGAATTCGTAACCCTCTTTCTATTTAAGATAC |
|  | mecW-R | AATGACGTGTTATTACAAGCATTATTGATTTAATC |
| Cloning of *psm-mec* | S2-XbaI | TCTTCTAGAATTCACTTGTCTTAAACTTTGTAGAAAAAGAAG |
|  | S3-SacI | GAGGAGCTCTATTTTATTTTCCATAATTGCCTACCCCATAAG |
| Deletion of *psm-mec* | psm-mec-U-F | TTGCATTCGGATTAAACTGG |
|  | psm-mec-U-R | GTTCGCTAGATAGGGGTCCCTCAAACAAATAAAAAATGTTAAAAATTCC |
|  | psm-mec-D-F | ATCACCTCAAATGGTTCGCTCAACTAAAAAACAGAGGAAATATTC |
|  | psm-mec-D-R | TTAATGGCATTCGACCAAAA |
|  | tetL-F | AGCGAACCATTTGAGGTGATCAACAAACGGGCCATATTG |
|  | tetL-R | GGGACCCCTATCTAGCGAACTTGAACTCTCTCCCAAAGTTGA |
|  | phleo-F | AGCGAACCATTTGAGGTGATGGATCCAATAGACCAGTTGC |
|  | phleo-R | GGGACCCCTATCTAGCGAACCGATTGCTGAACAGATTAATAA |
| Disruption of *rnc* | rnc-F | ACGAATTCAACAAGCATTTTCGCATTC |
|  | rnc-R | GTGGATCCTGGTGCACATATTCTTGG |
| Overproduction of AgrA | agrA-HisC-F | TAATAAGATAATAAAGTCAGTTAACGG |
|  | agrA-HisC-R | ATGATGATGATGATGATGTATTTTTTTAACGTTTCTCACCGATGC |
|  | AgrA-F-NdeI | CCATCATCATATGAAAATTTTCATTTGCGAAGACG |
|  | AgrA-R-BamHI | GGAGGATCCGTTAACTGACTTTATTATC |
| Detection of *mecA* | mecA-F | GGAGGATCCTCGTGTCAGATACATTTCGATTCA |
|  | mecA-R | AAGAAGCTTGTTGTAGCAGGAACACAAATGAATAAC |
| Circularized RACE | RACE-F | GACTTGCATTCAGGCTTTCG |
|  | RACE-R | TGCTTGTAATAACACCAGTGAAATC |
| Induction of *agrBDCA* | agrB-F | AAGAAGCTTAAATCGTATAATGACAGTGAGGAGAG |
|  | agrA-R2 | GGAGGATCCTTATATTTTTTTAACGTTTCTCACCGATG |

Adaptor sequence for cutting by restriction enzymes is underlined.
